# An EO771-Derived Metastatic Breast Cancer Model Exhibits RANKL-Associated Osteoimmune Remodeling Before Detectable Bone-Marrow Dissemination

**DOI:** 10.3390/biology15212014

**Published:** 2026-11-05

**Authors:** Diego Andrade Garcia, Maria Eduarda Globa Masset, Kauan Pinheiro da Silva Santos, Maria Alice Guerreiro, Gabriel Fidalgo, Marcos Vinicius Colaço, Liebert Parreiras Nogueira, Adriana Bonomo, Fabio Canto, Ana Carolina Monteiro

**Affiliations:** 1 Laboratory of Osteo and Tumor Immunology, Department of Immunobiology, Federal Fluminense University (UFF), Niteroi 24210-201, Brazil; diegodeandrad3@gmail.com (D.A.G.); mariaeduardamasset@gmail.com (M.E.G.M.); kp_santos@id.uff.br (K.P.d.S.S.); alice_guerreiro@id.uff.br (M.A.G.); 2 Graduate Program in Immunology and Inflammation, Federal University of Rio de Janeiro (UFRJ), Rio de Janeiro 21941-590, Brazil; fabiocanto@id.uff.br; 3 Laboratory of Applied Physics to Biomedical and Environmental Sciences, Physics Institute, State University of Rio de Janeiro, Rio de Janeiro 20550-900, Brazil; fidalgogqs@gmail.com (G.F.); mvcolaco@gmail.com (M.V.C.); 4 Oral Research Laboratory, Institute of Clinical Dentistry, University of Oslo, 0317 Oslo, Norway; l.p.nogueira@odont.uio.no; 5 Laboratory on Thymus Research, Oswaldo Cruz Institute, Rio de Janeiro 21040-900, Brazil; acbonomo@gmail.com; 6 Oswaldo Cruz Foundation (Fiocruz), Av. Brasil 4365, Manguinhos, Rio de Janeiro 21040-900, Brazil; 7 Research Network on Neuroinflammation (RENEURIN), Oswaldo Cruz Institute, Rio de Janeiro 21040-900, Brazil; 8 Laboratory of Immune Tolerance and Lymphocyte Homeostasis, Department of Immunobiology, Federal Fluminense University (UFF), Niteroi 24210-201, Brazil; 9 Department of Biochemistry and Immunology, Institute of Biological Sciences, Federal University of Minas Gerais (UFMG), Av. Antônio Carlos, 6627 - Pampulha ICB - Bloco K-4, sala 163, Belo Horizonte 31270-901, Brazil

**Keywords:** breast cancer, bone metastasis, osteoimmunology, RANKL, pre-metastatic niche, T cells, B cells, bone loss

## Abstract

Bone metastases are traditionally regarded as late manifestations of breast cancer progression. However, increasing evidence suggests that systemic skeletal alterations may emerge before overt tumor dissemination, raising the possibility that osteoimmune remodeling may contribute to the establishment of a permissive skeletal microenvironment. Pre-clinical studies from our group demonstrated that metastatic 4T1 breast cancer is associated with the expansion of RANKL-expressing adaptive immune populations and early skeletal alterations in BALB/c mice. Whether similar osteoimmune alterations occur in a distinct syngeneic immunocompetent breast cancer model remained unknown. Here, we generated an EO771-derived metastatic breast cancer population (EO771B) through serial multi-site in vivo selection under orthotopic, hematogenous and bone-associated pressures in immunocompetent C57BL/6 hosts. Compared with its parental counterpart, EO771B exhibited spontaneous dissemination to bone marrow, which was not detected for parental EO771 cells under the experimental conditions evaluated, while dissemination to tumor-draining lymph nodes (dLNs) and primary tumor growth did not differ significantly between the two populations. Microcomputed tomography revealed significant trabecular bone deterioration as early as day 11 following orthotopic inoculation, before bone-marrow dissemination became detectable by the clonogenic assay used in this study, with additional alterations in trabecular microarchitecture observed at later stages of tumor progression. These skeletal changes were accompanied by increased frequencies and/or numbers of RANKL-expressing CD4^+^ T cells and CD19^+^ B lymphocytes in the bone marrow, despite the absence of detectable tumor-derived colonies at the early time point. RANKL-expressing CD4^+^ T-cell populations also increased in tumor-dLNs and the primary tumor microenvironment during tumor progression. Adoptive transfer of tumor-educated leukocytes into lymphocyte-deficient *Rag1*^−/−^ recipients reproduced trabecular bone deterioration, demonstrating that tumor-educated leukocytes can functionally promote skeletal remodeling, although the specific contribution of lymphocyte-derived RANKL was not directly tested. Collectively, these findings demonstrate that EO771B tumor progression is associated with osteoimmune alterations characterized by increased RANKL-expressing lymphocyte populations and trabecular bone deterioration before bone-marrow dissemination becomes detectable by clonogenic assay. Together with our previous findings in a distinct syngeneic breast cancer model, these results support the occurrence of tumor-associated osteoimmune remodeling across different tumor–host systems and establish the EO771B–C57BL/6 model as a platform for further mechanistic investigation.

## Simple Summary

Breast cancer cells can spread to the skeleton, but changes in bone may begin before tumor cells become detectable in the bone marrow. In this study, we developed an EO771-derived breast cancer model in immunocompetent C57BL/6 mice that displays spontaneous dissemination to the bone marrow. Changes in trabecular bone structure were detected at an early stage of tumor progression, before tumor-cell dissemination to the bone marrow became detectable by the clonogenic assay used in this study. These skeletal changes were accompanied by increased RANKL expression in T and B lymphocytes in tumor-associated tissues and bone marrow. Furthermore, transfer of leukocytes from tumor-bearing mice reproduced trabecular bone deterioration in recipient mice, indicating that tumor-educated immune cells can contribute to skeletal remodeling. These findings establish the EO771B-C57BL/6 system as a model for investigating immune and skeletal changes associated with breast cancer progression and provide a basis for future studies of the mechanisms linking tumor-associated immune responses to the skeletal microenvironment.

## 1. Introduction

Bone metastases have traditionally been regarded as late manifestations of breast cancer progression, becoming clinically evident after systemic dissemination and establishment of overt skeletal lesions, a concept largely supported by the detection of bone involvement in advanced stages of disease and by the clinical burden associated with metastatic recurrence [1,2,3]. However, accumulating epidemiological and clinical evidence suggests that disturbances in skeletal homeostasis may emerge considerably earlier during breast cancer evolution [4,5,6,7,8]. Alterations in bone mineral density (BMD), increased prevalence of osteopenia and osteoporosis, higher fracture susceptibility, and changes in bone turnover have been reported across distinct breast cancer populations, including newly diagnosed patients without clinically detectable skeletal metastases and before prolonged exposure to systemic therapies [4,5,6,7,8,9]. Nevertheless, these findings remain heterogeneous and sometimes paradoxical, with studies reporting both increased and decreased BMD at diagnosis depending on menopausal status, ethnicity, and clinical context [10]. Collectively, these observations suggest that static bone-mass measurements alone may inadequately reflect dynamic biological processes within the skeletal microenvironment during tumor progression.

Bone remodeling is a homeostatic process continuously regulated by hormonal, mechanical, inflammatory, and immune-derived signals that coordinate osteoblast and osteoclast activity [7,11,12]. In the context of cancer, disruption of this tightly regulated balance may alter the cellular and molecular composition of the skeletal microenvironment, potentially influencing its susceptibility to subsequent metastatic colonization. Among the mechanisms that link systemic tumor progression to skeletal remodeling, immune-mediated regulation of bone homeostasis is particularly relevant [13,14]. Osteoimmunology has established extensive functional interactions between immune and skeletal cells, through which activated immune populations can directly influence osteoclastogenesis and bone resorption [15,16]. Among the molecular pathways governing these interactions, the RANK-RANKL-OPG axis occupies a central role by coordinating osteoclast differentiation, bone resorption, lymphoid organ development, and adaptive immune responses [17,18,19,20,21]. Sustained inflammatory signaling and persistent production of osteoclastogenic mediators, including RANKL, have been implicated in pathological bone remodeling across chronic inflammatory disorders and malignant conditions [22,23,24]. Whether tumor-associated adaptive immune responses engage these osteoimmune pathways early enough to modify the skeletal microenvironment before metastatic dissemination, however, remains incompletely understood in breast cancer. Importantly, our recent findings in human breast cancer showed that OPG-producing B cells and RANKL-expressing T cells define immune signatures associated with the subsequent development of bone metastases, supporting the clinical relevance of these osteoimmune populations [25].

Exploring breast cancer progression in immunocompetent mice, our group previously demonstrated that skeletal alterations may emerge before overt bone metastases rather than solely as consequences of established skeletal colonization [26]. Experimental evidence obtained by the orthotopic injection of the metastatic 4T1 breast tumor into BALB/c mice showed that expansion of tumor-associated RANKL-producing adaptive immune populations, particularly CD4^+^ T lymphocytes, precedes detectable bone colonization and promotes early osteolytic alterations through enhanced osteoclastogenic activity, contributing to pre-metastatic niche formation and subsequent bone metastasis establishment [26,27]. Conversely, studies using as a model the orthotopic inoculation of non-metastatic 67NR sibling tumor cells restricted to the mammary site are also capable of inducing systemic skeletal alterations despite the absence of local invasion or distant dissemination [28]. Rather than promoting bone loss, in situ 67NR tumors induced increased trabecular skeletal mass associated with expansion of IFN-γ^+^ IL-10^+^ CD8^+^ T cells and suppression of osteoclastogenic activity within the bone marrow microenvironment [28]. Moreover, recent evidence demonstrated enrichment of OPG-producing B cells in non-metastatic settings, which actively suppress osteoclastogenesis and inhibit bone metastatic progression [25]. Together, these findings indicate that tumor-induced adaptive immune remodeling may elicit either osteolytic or osteoprotective systemic responses depending on tumor biological behavior. Thus, rather than reflecting a uniform response to tumor burden, osteoimmune remodeling may differentially condition the skeletal microenvironment toward permissive or restrictive states for subsequent metastatic colonization.

Despite the evidence supporting immune-mediated modulation of bone remodeling in breast cancer models, it remains important to determine whether similar osteoimmune alterations occur in distinct syngeneic tumor–host systems. Experimental models that combine spontaneous bone-marrow dissemination with an intact immune system are particularly valuable for investigating the temporal relationship among tumor progression, adaptive immune responses, and skeletal remodeling. In this context, the EO771 mammary cancer cell line provides a syngeneic model for studies in immunocompetent C57BL/6 mice [29]. The C57BL/6 background offers the additional advantage of the broad availability of genetically modified and transgenic mouse strains that can subsequently be used for mechanistic investigation. To address this experimental need, we generated an EO771-derived metastatic breast cancer population (EO771B) through serial multi-site in vivo selection under orthotopic, hematogenous and bone-associated pressures in syngeneic immunocompetent C57BL/6 mice. Our objective was to establish a C57BL/6 model displaying spontaneous bone-marrow dissemination and to investigate whether osteoimmune alterations previously identified in other experimental systems also occur during EO771B tumor progression. We therefore examined the temporal relationship among tumor progression, adaptive immune alterations, skeletal remodeling, and detectable tumor-cell dissemination to the bone marrow.

## 2. Materials and Methods

### 2.1. Ethical Approval and Animals

All animal procedures were conducted in accordance with the guidelines established by the Brazilian National Council for the Control of Animal Experimentation (CONCEA) and approved by the Ethics Committee on Animal Use of Fluminense Federal University (CEUA/UFF; protocol CEUA 1723070324, ID 002093). Female C57BL/6 wild-type (WT) and *Rag1*-deficient (*Rag1*^−/−^) mice on the C57BL/6 background, aged 8–12 weeks and weighing 22–25 g, were obtained from the institutional animal facility and maintained under specific pathogen-free conditions with ad libitum access to food and water. Animals underwent an acclimatization period of approximately one week prior to experiments. No animal losses occurred before the predefined experimental endpoints, and all animals completing the experiments were included in the corresponding analyses. Investigators were aware of group allocation during the conduct of the animal experiments, whereas MicroCT reconstruction and quantitative analyses were performed under blinded conditions. Animals were monitored daily, and predefined humane endpoints included a maximum tumor volume of 1 cm^3^, loss of ≥5% of initial body weight, or deterioration in clinical condition. No animal reached these humane endpoints before the predefined experimental endpoints. Group sizes were determined based on prior experience with similar experimental models and expected biological variability, rather than formal statistical power calculations. Experimental group sizes generally ranged from 2 to 6 animals, as specified for each experiment and in the corresponding figure legends. The experimental unit was the individual animal. No animals were excluded from the completed experiments. Individual measurements that did not meet technical quality criteria were excluded from the corresponding analyses. The number of animals contributing to each analysis is indicated in the respective figure legends.

### 2.2. Cell Culture

Parental EO771 murine mammary carcinoma cells were commercially provided by ATCC (Manassas, VA, USA; CRL-3461). Both parental EO771 cells and the derived metastatic EO771B variant were maintained under sterile conditions in Dulbecco’s Modified Eagle Medium (DMEM, High Glucose 4500 mg/L, Gibco, Grand Island, NY, USA, 10569010) supplemented with 10% fetal bovine serum (FBS; Nova Biotecnologia, Cotia, Brazil; 10-bio500), 100 U/mL penicillin + 100 μg/mL streptomycin (Nova Biotecnologia; BR30111-01) and 10 mM HEPES buffer (Nova Biotecnologia; BR30237-01). Cells were cultured at 37 °C in a humidified atmosphere containing 5% CO_2_ and routinely passaged upon reaching approximately 80–90% confluence. Adherent cells were detached using trypsin-EDTA solution (Gibco; 25200-056), centrifuged and expanded in tissue culture flasks (Techno Plastic Products™, Trasadingen, Switzerland, TPP, 75 cm^2^ 90076; 150 cm^2^ 90151) according to experimental requirements. All cell lines used in this study were authenticated and regularly tested for Mycoplasma contamination using the MycoAlert Mycoplasma Detection Kit (Lonza, Basel, Switzerland, LT07-118) performed in-house.

### 2.3. Generation of the EO771-Derived Metastatic Variant (EO771B)

To generate an EO771-derived metastatic breast cancer population, parental EO771 cells were subjected to serial multi-site in vivo selection designed to apply selective pressures associated with primary tumor growth, hematogenous dissemination, and adaptation to the bone microenvironment. Female C57BL/6 mice (8–12 weeks old) simultaneously received 2.5 × 10^5^ EO771 cells by each of three inoculation routes: orthotopic injection into the fourth mammary fat pad, intravenous administration via the retro-orbital plexus and intratibial inoculation. Animals were anesthetized by intraperitoneal administration of ketamine (90 mg/kg) and xylazine (10 mg/kg) (Sigma Aldrich, St. Louis, MO, USA; 61763-23-3) before the corresponding experimental procedures. Each selection cycle included 5–8 mice. Twenty-five days after inoculation, mice were euthanized and cells were recovered from primary tumors, draining lymph nodes, lungs, liver, spleen and tibia-derived bone marrow. Solid organ-derived cell suspensions were obtained by mechanical dissociation, whereas bone samples were additionally subjected to enzymatic digestion. Cells isolated from each anatomical site were expanded independently under the standard culture conditions described above. Tumor-cell populations recovered from the different anatomical sites were subsequently pooled, expanded in vitro and reinoculated into new recipient C57BL/6 mice using the same three administration routes. This sequential process of recovery, expansion, pooling and reinoculation was repeated for three consecutive cycles to select for tumor cells capable of surviving and growing across these anatomical compartments. Following the third selection cycle, pooled EO771-derived cells were inoculated exclusively into the fourth mammary fat pad (5 × 10^5^ cells/mouse) of C57BL/6 hosts to evaluate tumor-cell dissemination from the primary tumor (Supplementary Figure S1). Animals were euthanized 35 days later for assessment of spontaneous metastatic dissemination. The resulting selected EO771-derived population was designated EO771B.

### 2.4. Orthotopic Tumor Induction and Monitoring

Female C57BL/6 mice received orthotopic inoculation of EO771 or EO771B cells (5 × 10^5^ cells) suspended in 100 μL 1X PBS (Gibco, 10010023) into the fourth mammary fat pad. Tumor-bearing animals were monitored throughout the experimental period and euthanized at predefined experimental endpoints according to the corresponding experimental series and downstream analyses. Early-stage analyses were performed at day 10 or 11, whereas later-stage analyses were performed at day 25, 30, 32 or 35, as specified for each individual experiment in the corresponding Methods subsection and figure legend. Primary tumor growth was assessed longitudinally using digital calipers. Tumor dimensions were measured in three axes, and tumor volume was estimated as V=a×b×c, where *a*, *b* and *c* correspond to tumor height, width and depth, respectively. At euthanasia, primary tumors were surgically excised and weighed for determination of final tumor burden.

### 2.5. Assessment of Disseminated Tumor Cells by Clonogenic Assay

Disseminated tumor cells were evaluated by clonogenic assays performed using cells recovered from secondary organs of EO771B tumor-bearing mice. Inguinal and popliteal tumor-draining lymph nodes (dLNs) were collected from each mouse and pooled to increase cell recovery for clonogenic analysis. dLNs were mechanically dissociated to obtain single-cell suspensions, whereas iliac bone-derived marrow samples were additionally subjected to enzymatic digestion (1 mg/mL collagenase I, Gibco 17100-017 and DNase, 0.1 mg/mL—Worthington LS002138, Lakewood, NJ, USA). Cell suspensions were collected in complete DMEM medium (Gibco), quantified and plated in 6-well plates (Techno Plastic Products™, TPP, Z707759) using previously standardized serial dilutions. Cultures were maintained under standard conditions (37 °C, 5% CO_2_) for 14 days to allow the survival and expansion of clonogenic tumor-derived cells under the culture conditions used. At the end of the incubation period, wells were washed with 1X PBS (Gibco, 10010023), fixed with 100% methanol (Sigma-Aldrich, Burlington, MA, USA, 67-56-1) and stained with 0.3% methylene blue (Sigma-Aldrich, 61-73-4). Clonogenic foci were quantified macroscopically as stained colonies. The assay was used to assess detectable spontaneous tumor-cell dissemination to dLNs and bone marrow following orthotopic EO771B tumor growth. Accordingly, the detection of clonogenic colonies was interpreted as evidence of tumor-cell dissemination to these tissues rather than as histological evidence of established metastatic lesions. During previous assay standardization, serial dilutions of total cells recovered from the analyzed organs (10^6^, 10^5^, 10^4^, 10^3^, 10^2^, and 10^1^ cells per well) were evaluated in six-well plates. Clonogenic tumor colonies could be detected in wells seeded with as few as 10^2^ total organ-derived cells. Since an individual colony may originate from a single clonogenic tumor cell or a small cluster of tumor cells, colony formation was interpreted as evidence of viable clonogenic tumor cells rather than as a direct measure of the number of disseminated tumor cells.

### 2.6. Micro-CT Scanning, Image Reconstruction, Data Processing and Analysis

Soft tissues were excised from the iliac bones of C57BL/6 mice. The bones were then fixed with 100% ethanol at 4 °C. Micro-computed tomography (MicroCT) scans were conducted using Bruker Skyscan 1172 (Kontich, Belgium). Each sample was wrapped in moist gauze, placed inside an Eppendorf^®^ tube (Eppendorf SE, Hamburg, Germany) and scanned at 65 kV and 143 μA, with a rotation step of 0.59°, 2 × 2 pixel binning, and an exposure time of 0.63 s per frame (three frames per projection). The resulting voxel size was 5.0 μm per pixel. To minimize low-energy X-ray interference, a 0.5 mm aluminum filter was employed. Micro-CT image reconstruction was performed using the NRecon software (version 1.7.5, Bruker, Bruker microCT, Kontich, Belgium), applying a beam hardening correction of 40%, ring artifact correction of 8, and no smoothing. The samples were consistently oriented to ensure reproducibility and uniformity across all scans. Before MicroCT analysis, the elongated unbranched portion of the iliac bone was removed, and the remaining V-shaped bifurcated portion was used for analysis. The volume of interest (VOI) was defined within the trabecular compartment of this V-shaped region and standardized across samples using the same anatomical boundaries. The trabecular bone was digitally segmented from the cortical bone using the CTAn software (version 1.20.3, Bruker), and morphometric parameters were calculated following standardized guidelines for rodent bone microarchitecture analysis. The analyses included trabecular bone volume fraction (BV/TV), bone surface (BS), trabecular number (Tb.N), thickness (Tb.Th), and separation (Tb.Sp). All reconstructions and analyses were conducted under blinded conditions. For each sample, 200 slices corresponding to a height of 1 mm were selected from the designated region. A task list in the CTAn facilitated the separation of cortical and trabecular areas, segmentation, and quantification of the analyzed parameters.

### 2.7. Flow Cytometry Analysis

To characterize adaptive immune populations during tumor progression, female C57BL/6 mice were euthanized at early and later stages following orthotopic EO771B inoculation, with the actual experimental day specified for each analysis in the corresponding figure legend. Tumor-draining lymph nodes (dLNs), tumor-infiltrating lymphocytes (TILs) and bone marrow (BM) from the iliac bones and femurs were collected for immunophenotypic analysis. Inguinal and popliteal dLNs from each animal were pooled to increase cell recovery for flow-cytometric analyses. Single-cell suspensions from dLNs were obtained by mechanical dissociation using syringe plungers followed by filtration through 70 μm nylon strainers. Tumor tissues were mechanically fragmented and processed to isolate TILs (Tumor Dissociation kit-mouse, 130-096-730, Miltenyi Biotec, Bergisch Gladbach, Germany), according to the manufacturer’s recommendations. For BM analyses, cells recovered from the iliac bones and femurs of each animal were combined to increase cell recovery, particularly for T-cell analyses. Cell suspensions were centrifuged and resuspended in PBS before staining procedures. Cell viability was assessed using Fixable Viability Stain (FVS510) diluted in PBS and incubated for 30 min at 4 °C. Following viability staining, Fc receptors were blocked using anti-mouse CD16/CD32 antibody for 15 min at 4 °C to minimize nonspecific binding. Surface staining was subsequently performed using fluorochrome-conjugated monoclonal antibodies against CD3, CD4, CD8 and CD19 to identify T and B lymphocytes. Cells were incubated with antibody cocktails diluted in flow-cytometer cell (FACS) buffer containing 1X PBS (Gibco, 10010023), fetal bovine serum (FBS, Nova Biotecnologia 10-bio500) and EDTA (Sigma Aldrich, E9884) for 45 min at 4 °C in the dark. After surface staining, cells were fixed and permeabilized using the eBioscience™ Foxp3/Transcription Factor Staining Buffer Set (Thermo Fisher Scientific, San Diego, CA, USA) (00-5523-00), according to the manufacturer’s recommendations. Intracellular staining was then performed using monoclonal antibodies against Foxp3 and RANKL. Cells were incubated with intracellular antibody cocktails for 60 min at 4 °C, washed twice with permeabilization buffer and resuspended in PBS for acquisition. Samples were acquired using an LSRFortessa X-20 flow cytometer (BD Biosciences, Franklin Lakes, NJ, USA) at the Flow Cytometry Core Facility of the Federal University of Rio de Janeiro. The total number of available events from each sample was acquired rather than a predefined fixed number of events. Fluorescence-minus-one (FMO) controls, containing all antibodies in the corresponding staining panel except the antibody corresponding to the marker being evaluated, were used to assist in defining positivity gates. Data analysis was performed using FlowJo software (version 10.8.1; BD Life Sciences, Ashland, OR, USA). After exclusion of debris, doublets, and non-viable cells, lymphocyte populations were identified according to the corresponding lineage markers. Absolute cell numbers, when reported, were calculated by multiplying the frequency of the corresponding population by the total number of cells recovered from each individual sample. Representative gating strategies are shown in Supplementary Figures S2 and S3. Monoclonal antibodies and fluorochromes employed in flow cytometric analyses are summarized in Supplementary Table S1.

### 2.8. Preparation of Soluble EO771B Tumor Antigen (sAg)

Primary EO771B tumors were collected from orthotopically inoculated C57BL/6 mice following euthanasia. Under sterile conditions, tumor tissues were mechanically fragmented and subjected to repeated freeze–thaw cycles consisting of immersion in liquid nitrogen followed by thawing at 37 °C until complete cellular disruption was achieved, as indicated by increased suspension turbidity. The resulting lysates were centrifuged at 12,000 rpm for 5 min and supernatants were collected and filtered through 0.22 μm membranes to remove cellular debris. Total protein concentration of the soluble antigen preparation (sAg) was quantified by spectrophotometry at 280 nm. Soluble antigen aliquots were stored at −20 °C until use in adoptive transfer protocols.

### 2.9. Adoptive Transfer Experiments

Female C57BL/6 donor mice received orthotopic inoculation of EO771B cells (5 × 10^5^ cells) into the fourth mammary fat pad. Eleven days following inoculation, at an early stage of tumor progression at which bone-marrow dissemination was not detectable by the clonogenic assay used in this study, donor animals were euthanized and dLNs (inguinal and popliteal) were collected. Inguinal and popliteal dLNs from each donor were pooled for cell recovery. Adoptive transfer experiments were performed using total leucocytes derived from tumor-dLNs of EO771B-bearing mice or from tumor-free naïve C57BL/6 mice, as controls. Single-cell suspensions were generated by mechanical dissociation using nylon strainers (BD Biosciences). Aliquots containing 1 × 10^6^ lymph node-derived leukocytes were centrifuged and resuspended in 100 μL 1X PBS (Gibco, 10010023) supplemented with soluble EO771B tumor antigen (sAg; 25 μg/animal). Recipient animals consisted of immunodeficient *Rag1*^−/−^ mice and immunocompetent wild-type C57BL/6 mice (WT). Prior to adoptive transfer, recipients were anesthetized with ketamine (90 mg/kg) and xylazine (10 mg/kg) (Sigma Aldrich) administered intraperitoneally (i.p.). Prepared cell suspensions were transferred intravenously through the retro-orbital plexus into recipient mice (3–4 animals/group). Eighteen days following adoptive transfer, recipient animals were euthanized and iliac bones were collected for MicroCT analyses.

### 2.10. Statistical Analysis

Quantitative data are presented as mean ± standard deviation (SD), unless otherwise indicated in the figure legends. Statistical analyses were performed using GraphPad Prism software (version 8.0.1; GraphPad Software, San Diego, CA, USA). Statistical tests were selected according to the experimental design and number of groups being compared, as specified in the corresponding figure legends. Longitudinal tumor-growth data were analyzed using two-way repeated-measures ANOVA, considering group and time as factors, followed by the multiple-comparison procedure specified in the corresponding figure legend. Comparisons involving more than two independent groups, including the adoptive transfer experiments, were performed using one-way ANOVA followed by Tukey’s multiple comparisons test. The resulting *p* values were adjusted for multiple comparisons using Tukey’s procedure. Comparisons between two independent groups were performed using unpaired two-tailed Student’s *t*-tests. For the analyses of trabecular bone microarchitecture and lymphocyte populations, comparisons were selected according to the experimental objectives. Naïve and EO771B-bearing mice were compared at D11 and D25 to investigate tumor-associated alterations during the early and later stages of tumor progression, respectively. Comparisons between independent D11 and D25 groups were performed to assess additional changes during tumor progression. Within the primary tumor microenvironment, comparisons were restricted to D11 and D25. Relative frequencies and absolute cell numbers of lymphocyte populations were evaluated as complementary outcomes. These biologically defined comparisons were analyzed using unpaired two-tailed Student’s *t*-tests, and nominal *p* values are reported without adjustment for multiple comparisons. Given the relatively small number of animals per experimental group (generally 3–6 mice/group), formal normality testing was not systematically performed, as such tests have limited statistical power with small sample sizes. Non-parametric tests were used when appropriate for the experimental design and data characteristics, as specified in the corresponding figure legends. Group sizes were determined based on previous experience with similar metastatic breast cancer models rather than formal power calculations. Individual measurements that did not meet technical quality criteria were excluded from the corresponding analyses. These exclusions were considered independently of the statistical significance of the results. Statistical significance was defined as *p* < 0.05. Significance levels are represented as follows: *p* < 0.05 (*); *p* < 0.01 (**); *p* < 0.001 (***); *p* < 0.0001 (****)**,** and ns: not significant. For analyses involving Tukey’s multiple comparisons test, significance annotations correspond to adjusted *p* values; for the hypothesis-driven pairwise comparisons, they correspond to nominal *p* values.

## 3. Results

### 3.1. Serial Multi-Site In Vivo Selection Generates an EO771-Derived Population with Bone-Marrow Dissemination

To establish an EO771-derived breast cancer population capable of spontaneous dissemination to the bone marrow (BM) in immunocompetent hosts, parental EO771 cells were subjected to serial in vivo selection under orthotopic, hematogenous and bone-associated selective pressures in female C57BL/6 mice (Supplementary Figure S1). Briefly, parental cells were inoculated through orthotopic, intravenous and intratibial routes, followed by recovery of surviving populations from primary tumors, dLNs, lungs, liver, spleen and tibia-derived BM (Supplementary Figure S1). Recovered populations were independently expanded, pooled and reinoculated into female C57BL/6 recipient mice for three consecutive cycles, generating the EO771-derived metastatic population termed EO771B (Supplementary Figure S1). After completing the selection protocol, EO771B cells were inoculated exclusively via the orthotopic route to evaluate spontaneous tumor-cell dissemination from the primary tumor (Supplementary Figure S1).

**Figure 1:**
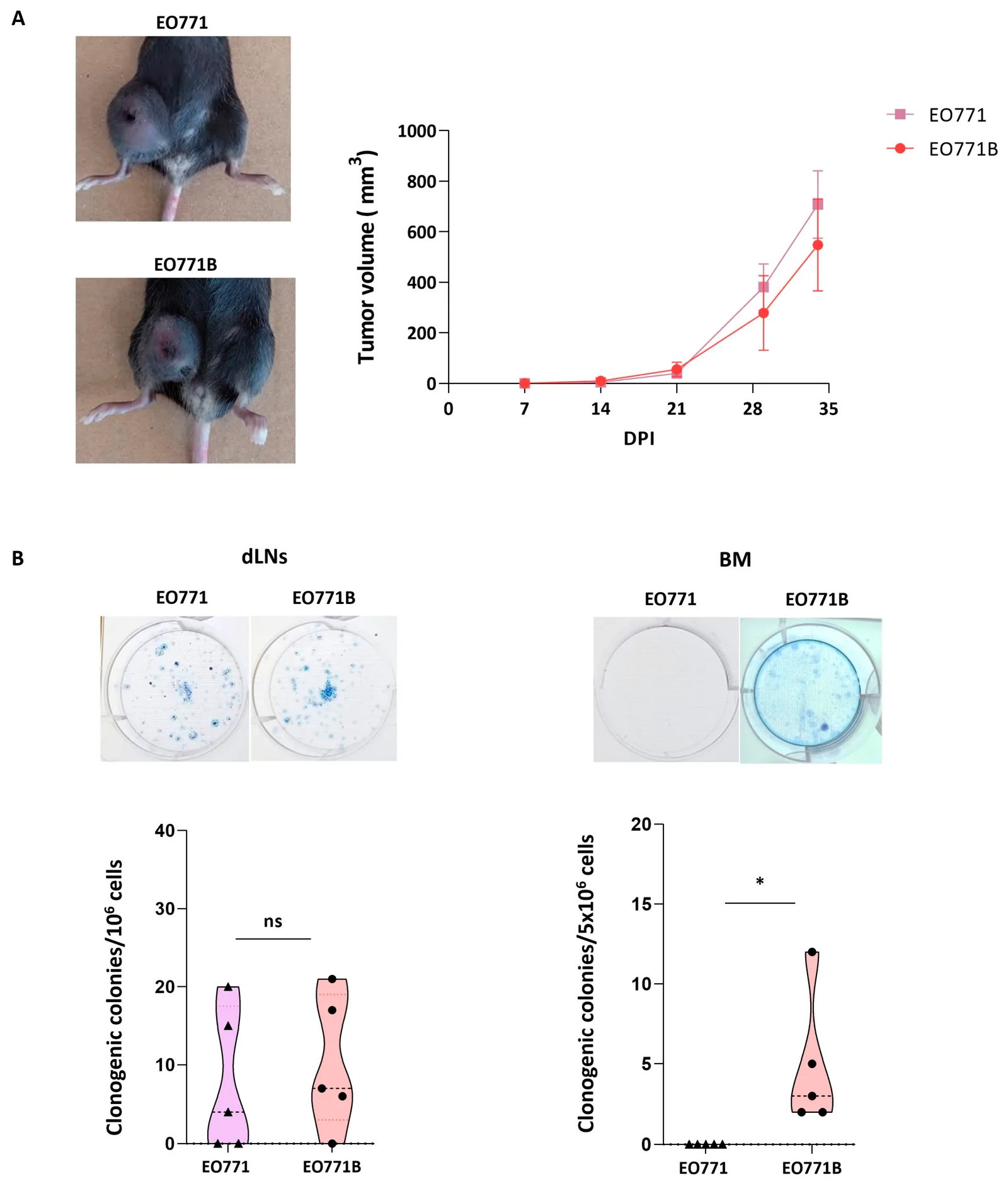
**In vivo characterization of parental EO771 and the selected EO771B breast cancer population.** (**A**) Representative images of mammary tumors and longitudinal tumor growth following orthotopic inoculation of parental EO771 or EO771B cells. Tumor volume was monitored up to day 35 post-inoculation (DPI). (**B**) Clonogenic tumor-cell burden in tumor-draining lymph nodes (dLNs) and bone marrow (BM) following orthotopic inoculation of parental EO771 or EO771B cells. Representative images of clonogenic cultures are shown above the corresponding quantitative analyses. Colony numbers are expressed relative to the number of cells plated, as indicated on the respective y-axes. Data are representative of three independent experiments with *n* = 6 mice per group. Tumor growth curves were analyzed using two-way repeated-measures ANOVA (**A**), whereas clonogenic colony counts were compared using an unpaired two-tailed Student’s *t*-test (**B**). * *p* < 0.05; ns, not significant.

Orthotopic inoculation of parental EO771 and EO771B cells resulted in progressive primary tumor growth, with no evident difference in tumor growth kinetics between the two populations (Figure 1A). Clonogenic assays demonstrated spontaneous tumor-cell dissemination to dLNs in both EO771- and EO771B-bearing mice, with no significant difference in clonogenic burden between the groups (Figure 1B). In contrast, clonogenic tumor-derived colonies were detected in the BM of EO771B-bearing mice, whereas no colonies were detected in the BM of mice bearing parental EO771 tumors under the experimental conditions evaluated (Figure 1B). Consistent with the comparable primary tumor growth, endpoint primary tumor weight did not differ between parental EO771- and EO771B-bearing animals (Supplementary Figure S4A). EO771B-bearing mice exhibited reduced spleen weight compared with animals bearing parental EO771 tumors (Supplementary Figure S4B). Despite these differences, total cellularity of dLNs and BM remained comparable between groups (Supplementary Figure S4C,D). At day 35, clonogenic tumor-cell dissemination to dLNs was detected in 60% of EO771-bearing and 80% of EO771B-bearing mice, whereas BM dissemination was detected in 100% of EO771B-bearing mice and was not detected in mice bearing parental EO771 tumors (Supplementary Figure S4E).

Collectively, these findings show that the serially selected EO771B population retains primary tumor growth comparable to parental EO771 cells while displaying spontaneous clonogenic tumor-cell dissemination to the BM. These characteristics establish the EO771B-C57BL/6 system as a model for investigating osteoimmune alterations associated with breast cancer progression and bone-marrow dissemination in an immunocompetent syngeneic host.

### 3.2. Early Skeletal Remodeling Precedes Detectable Bone-Marrow Dissemination During EO771B Tumor Progression

**Figure 2:**
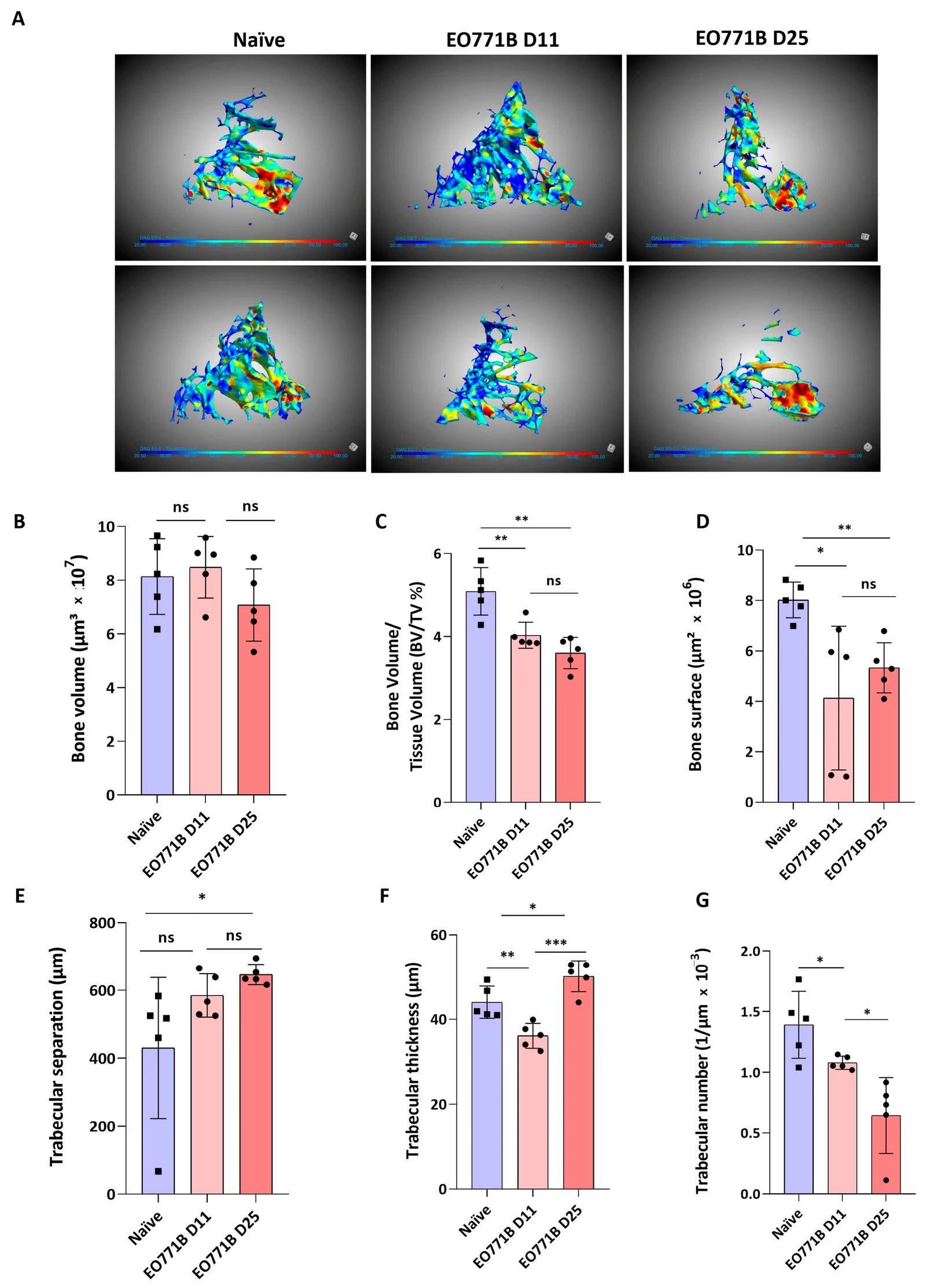
**Early trabecular bone deterioration precedes detectable bone marrow dissemination during EO771B tumor progression.** (**A**) Representative three-dimensional microcomputed tomography (MicroCT) reconstructions of iliac trabecular bone from tumor-free mice (Naïve) and EO771B-bearing mice at day 11 (D11) and day 25 (D25) following orthotopic tumor inoculation. The color gradient indicates local trabecular thickness, ranging from blue (thinnest, 20 µm) to red (thickest, 100 µm). Quantitative Micro-CT analysis of (**B**) bone volume (BV), (**C**) bone volume fraction (BV/TV), (**D**) bone surface area (BS), (**E**) trabecular separation (Tb.Sp), (**F**) trabecular thickness (Tb.Th), and (**G**) trabecular number (Tb.N). Data are presented as mean ± SD. Data were obtained from two independent experiments, with *n* = 5 mice per group. The Naïve group comprises tumor-free control mice corresponding to the D11 and D25 experimental series, which were pooled to provide the baseline reference group. Statistical analyses were performed using unpaired two-tailed Student’s *t*-tests, with nominal *p* values reported without adjustment for multiple comparisons. Statistical significance is indicated as follows: * *p* < 0.05; ** *p* < 0.01; *** *p* < 0.001; ns, not significant.

Given the spontaneous bone-marrow dissemination observed in the EO771B model, we next investigated whether skeletal alterations emerged during tumor progression before tumor-cell dissemination to the BM became detectable. Microcomputed tomography (MicroCT) analyses of iliac bones were performed at an early stage of tumor progression (day 11; D11) and at a later stage (day 25; D25) (Figure 2A; Supplementary Table S2). Independent clonogenic assays performed at closely corresponding early and later time points (D10 and D30, respectively) showed no detectable tumor-derived colonies in BM at the early time point, whereas clonogenic tumor-cell dissemination to BM was detected at the later time point (Supplementary Figure S5). Tumor-free mice (naïve) were included as controls to establish skeletal parameters.

At D11, EO771B-bearing mice already exhibited significant alterations in trabecular bone microarchitecture compared with naïve controls (Figure 2B–G; Supplementary Table S2). Early skeletal changes included significant reductions in BV/TV, BS, Tb.Th, and Tb.N, whereas BV and Tb.Sp did not differ significantly from naïve controls. By day 25, BV/TV, BS, Tb.Th and Tb.N remained significantly altered relative to naïve controls, while Tb.Sp was significantly increased as compared to the naïve group. Tb.Th also differed significantly between D11 and D25, indicating additional alterations in trabecular microarchitecture during tumor progression. No significant differences between D11 and D25 were detected for the remaining bone parameters (Figure 2B–G; Supplementary Table S2).

Collectively, these findings indicate that trabecular bone deterioration is already detectable at an early stage of EO771B tumor progression, before bone-marrow dissemination becomes detectable by the clonogenic assay used in this study (Supplementary Figure S5), with additional alterations in trabecular microarchitecture occurring at later stages of tumor progression.

### 3.3. EO771B Progression Is Associated with Compartment-Specific Remodeling of RANKL-Expressing Adaptive Immune Populations

To investigate whether EO771B progression is associated with alterations in adaptive immune populations during early and later stages of tumor progression, lymphocyte composition was evaluated in tumor-dLNs and within the primary tumor tissue at days 11 (D11) and 25 (D25) following orthotopic tumor inoculation. Initial analyses revealed changes in lymphocyte composition within dLNs (Supplementary Figure S6). EO771B-bearing mice exhibited reduced relative frequencies of total CD3^+^ T lymphocytes compared with tumor-free controls at both experimental time points, whereas absolute numbers did not differ significantly between groups (Supplementary Figure S6A–C). In contrast, total CD19^+^ B lymphocytes displayed increased frequencies and absolute numbers as early as day 11, a phenotype maintained at day 25 (Supplementary Figure S6I–K), indicating an expansion of the B-cell compartment in tumor-dLNs during EO771B progression.

Within the primary tumor microenvironment, the relative frequency of CD3^+^ T cells decreased from day 11 to day 25, whereas their absolute number increased over the same period (Supplementary Figure S7A–C). The frequency of CD19^+^ B cells was numerically higher at day 25 than at day 11, whereas their absolute number increased significantly at the later time point (Supplementary Figure S7I–K). The relative frequencies of CD4^+^ and CD8^+^ T-cell populations in dLNs remained comparable between naïve and EO771B-bearing mice at both time points, and no significant differences in their absolute numbers were detected (Supplementary Figure S6D–H).

Regulatory CD4^+^Foxp3^+^ T cells showed a compartment-dependent pattern. In dLNs, their frequency was increased in EO771B-bearing mice at day 25, whereas their absolute numbers were increased relative to naïve controls at both day 11 and day 25 (Supplementary Figure S8A–C). In the primary tumor, the frequency of CD4^+^Foxp3^+^ T cells remained stable between days 11 and 25, whereas their absolute number increased significantly at day 25 (Supplementary Figure S8D–F).

**Figure 3:**
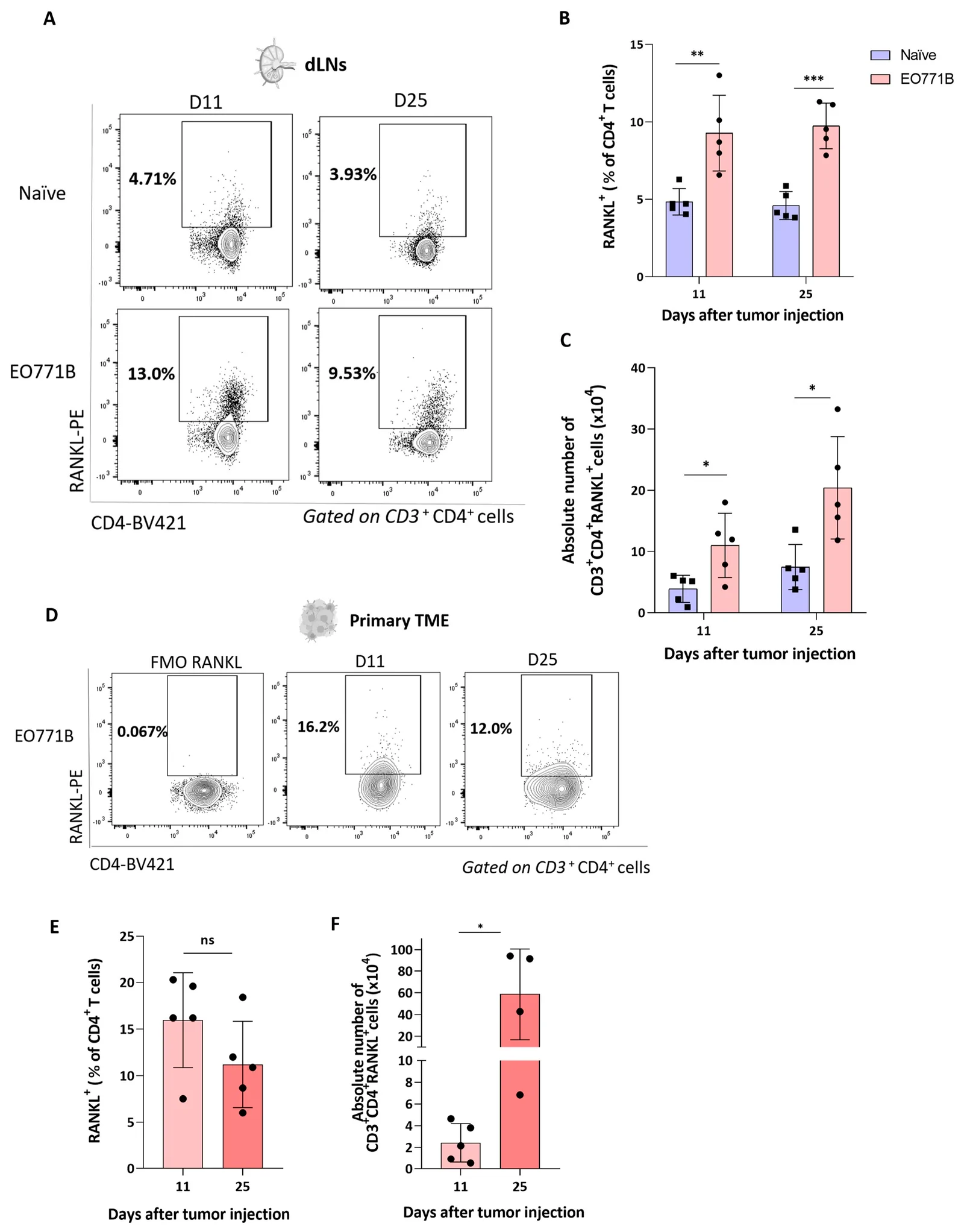
**RANKL-expressing CD4^+^ T-cell populations in tumor-dLNs and the primary tumor microenvironment during EO771B progression.** Representative flow cytometry plots and quantification of RANKL expression by CD4^+^ T lymphocytes in tumor-dLNs and the primary tumor microenvironment (TME) at days 11 (D11) and 25 (D25) following orthotopic EO771B inoculation. (**A**–**C**) Representative plots (**A**), frequency (**B**), and absolute number (**C**) of CD4^+^RANKL^+^ T cells in dLNs from naïve and EO771B-bearing mice. (**D**–**F**) Representative plots, including fluorescence-minus-one (FMO) control (**D**), frequency (**E**), and absolute number (**F**) of CD4^+^RANKL^+^ T cells within the primary TME at D11 and D25. Data are presented as mean ± SD. Data were obtained from two independent experiments, with *n* = 5 mice per group, except where individual technical exclusions apply. The indicated comparisons were selected according to the experimental objectives: naïve versus EO771B-bearing mice at each time point in dLNs and D11 versus D25 within the primary TME. Statistical analyses were performed using unpaired two-tailed Student’s *t*-tests, with nominal *p* values reported without adjustment for multiple comparisons. Statistical significance is indicated as follows: * *p* < 0.05; ** *p* < 0.01; *** *p* < 0.001; ns, not significant.

Given previous evidence implicating lymphocyte-derived RANKL in tumor-associated skeletal remodeling and metastatic progression [25,26], we next evaluated whether EO771B progression was associated with changes in RANKL-expressing adaptive immune populations. Analysis of tumor-dLNs demonstrated significant increases in both the frequency and absolute number of RANKL-expressing CD4^+^ T lymphocytes in EO771B-bearing mice compared with naïve controls at day 11, and these populations remained increased at day 25 (Figure 3A–C; Supplementary Table S3). RANKL-expressing CD4^+^ T cells were also readily detected within the primary tumor microenvironment, where their frequency did not differ significantly between days 11 and 25, whereas their absolute number was numerically higher at day 25, without reaching statistical significance (Figure 3D–F; Supplementary Table S3). In contrast, RANKL-expressing CD8^+^ T lymphocytes exhibited a distinct compartment-dependent pattern (Supplementary Figure S9). In dLNs, the frequency of RANKL-expressing CD8^+^ T cells was unchanged at day 11 but significantly increased at day 25 compared with naïve controls, whereas their absolute number did not differ significantly between groups at either time point (Supplementary Figure S9A–C). Within the primary tumor microenvironment, the frequency of RANKL-expressing CD8^+^ T cells increased significantly from day 11 to day 25, while their absolute number remained statistically unchanged over the same period (Supplementary Figure S9D–F).

**Figure 4:**
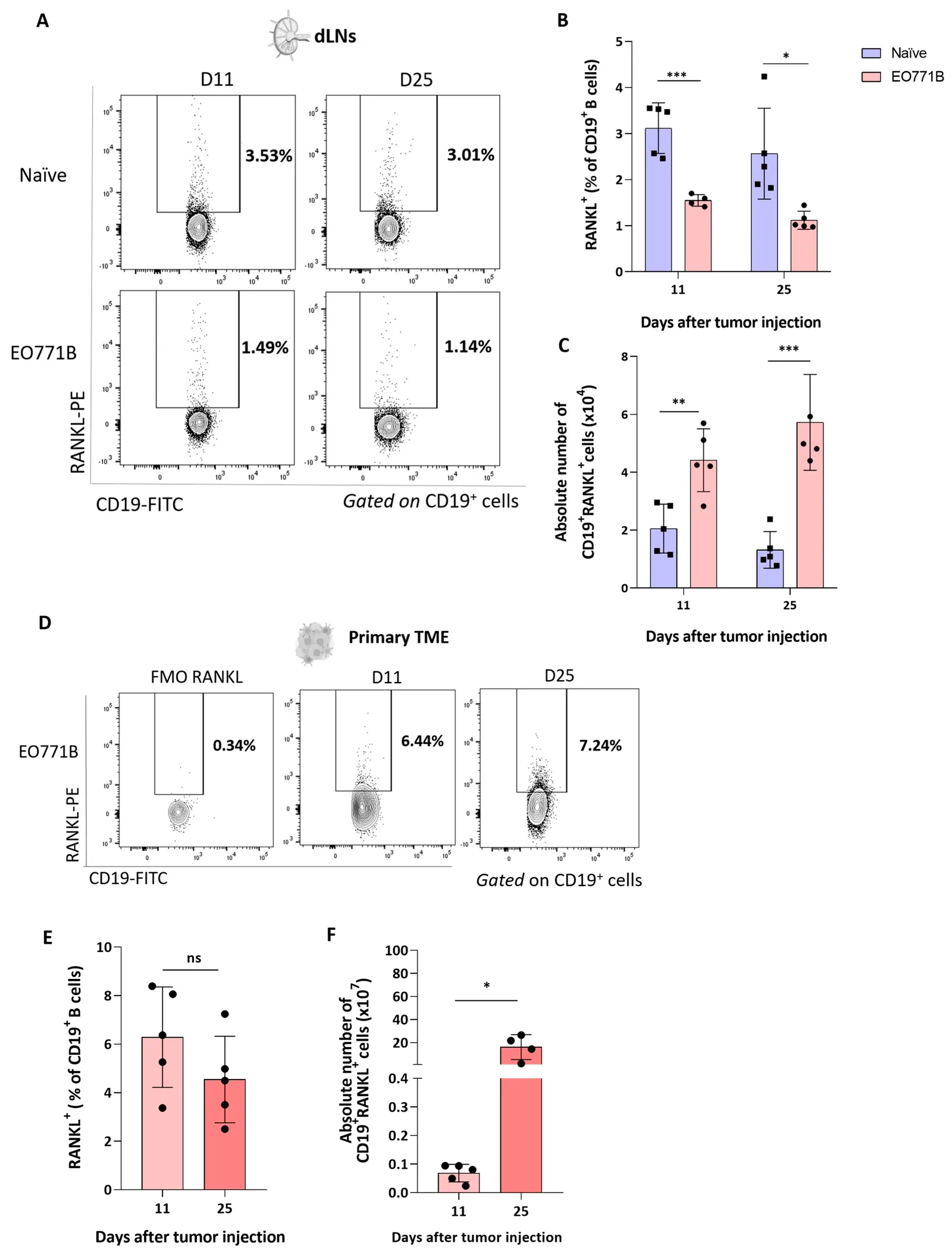
**RANKL-expressing CD19^+^ B-cell populations in tumor-dLNs and the primary tumor microenvironment during EO771B progression.** Representative flow cytometry plots and quantification of RANKL expression by CD19^+^ B lymphocytes in tumor-dLNs and the primary tumor microenvironment (TME) at days 11 (D11) and 25 (D25) following orthotopic EO771B inoculation. (**A**–**C**) Representative plots (**A**), frequency (**B**), and absolute number (**C**) of CD19^+^RANKL^+^ B cells in dLNs from naïve and EO771B-bearing mice. (**D**–**F**) Representative plots, including fluorescence-minus-one (FMO) control (**D**), frequency (**E**), and absolute number (**F**) of CD19^+^RANKL^+^ B cells within the primary TME at D11 and D25. Data are presented as mean ± SD. Data were obtained from two independent experiments, with *n* = 5 mice per group, except where individual technical exclusions apply. The indicated comparisons were selected according to the experimental objectives: naïve versus EO771B-bearing mice at each time point in dLNs and D11 versus D25 within the primary TME. Statistical analyses were performed using unpaired two-tailed Student’s *t*-tests, with nominal *p* values reported without adjustment for multiple comparisons. Statistical significance is indicated as follows: * *p* < 0.05; ** *p* < 0.01; *** *p* < 0.001; ns, not significant.

B cells also displayed EO771B-associated alterations in RANKL expression. Despite reduced frequencies of RANKL-expressing CD19^+^ B cells in dLNs at both time points, their absolute numbers significantly increased in EO771B-bearing mice compared with naïve controls, consistent with the increased absolute numbers of total B cells observed in dLNs (Figure 4A–C; Supplementary Table S3; Supplementary Figure S6I–K). RANKL-expressing CD19^+^ B cells were similarly detected within primary tumors, with no significant change in their frequency between days 11 and 25, whereas their absolute number increased significantly at day 25 (Figure 4D–F; Supplementary Table S3).

Collectively, these findings show that EO771B tumor progression is associated with early and compartment-specific remodeling of adaptive immune populations, including alterations in RANKL-expressing CD4^+^ T, CD8^+^ T, and B lymphocytes. Notably, increased frequencies and absolute numbers of RANKL-expressing CD4^+^ T cells, together with increased absolute numbers of RANKL-expressing CD19^+^ B cells, were already evident in dLNs at D11, an early stage at which BM dissemination was not yet detectable in the clonogenic assays performed at the corresponding early experimental window.

### 3.4. Early Enrichment of RANKL-Expressing Lymphocytes in Bone Marrow Precedes Detectable Tumor-Cell Dissemination

To determine whether EO771B-associated osteoimmune alterations extended to the bone marrow during early tumor progression, immune populations within BM were evaluated at days 11 (D11) and 25 (D25) following orthotopic tumor inoculation. Initial analyses demonstrated that EO771B-bearing mice exhibited increased absolute numbers of total CD3^+^ T and CD19^+^ B lymphocytes in BM at day 11, without significant changes in their relative frequencies (Supplementary Figure S10A–C,I–K). These increases were no longer observed at day 25. Similarly, the relative frequencies of CD4^+^ and CD8^+^ T lymphocytes did not differ significantly between naïve and EO771B-bearing mice, whereas their absolute numbers were increased at day 11 and were no longer significantly different from naïve controls at day 25 (Supplementary Figure S10D–H). Thus, EO771B progression was associated with an early increase in the absolute abundance of multiple adaptive immune populations within BM.

**Figure 5:**
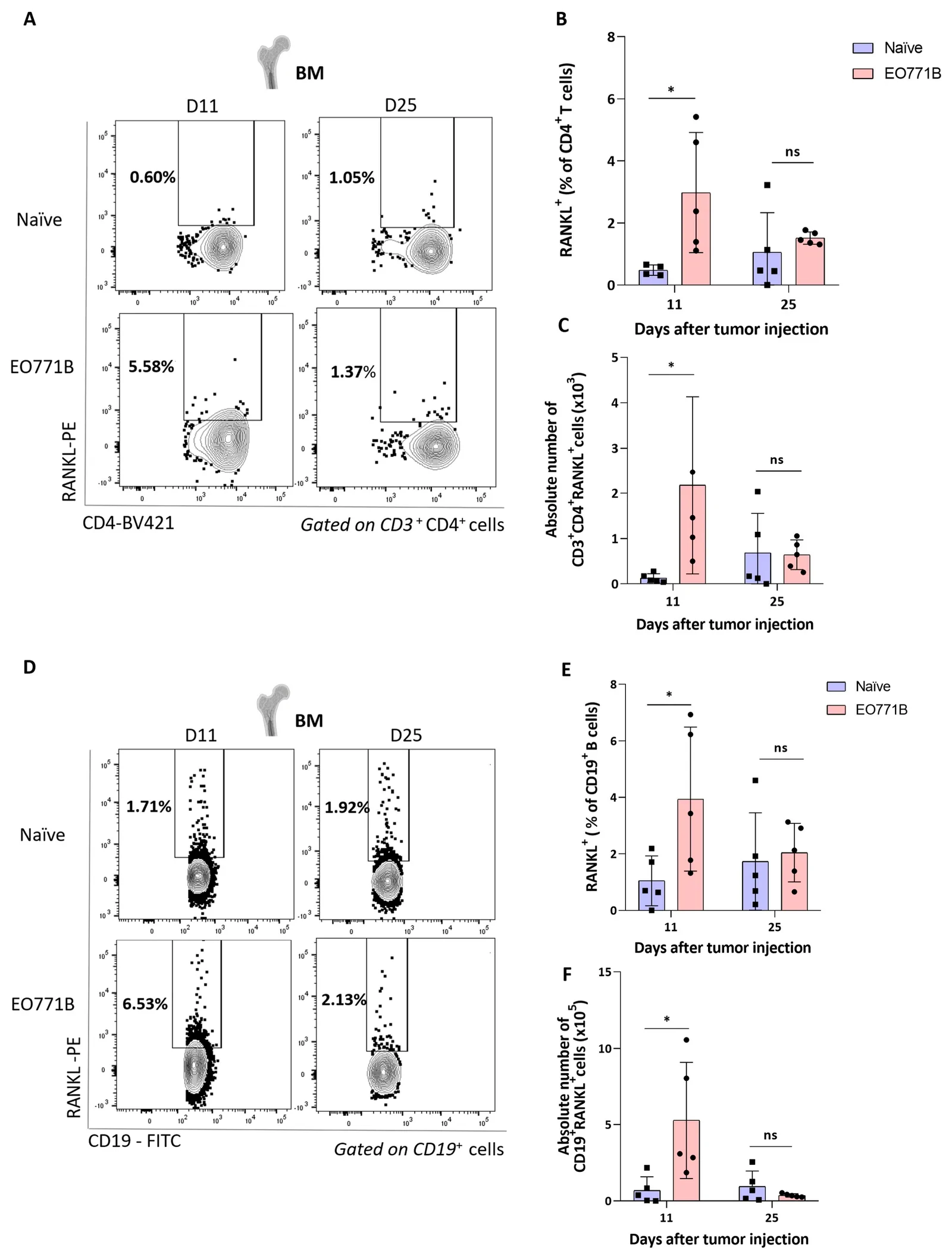
**Early enrichment of RANKL-expressing CD4^+^ T and CD19^+^ B lymphocytes in bone marrow during EO771B progression.** Representative flow cytometry plots and quantification of RANKL expression by CD4^+^ T and CD19^+^ B lymphocytes in bone marrow (BM) from naïve and EO771B-bearing mice at days 11 (D11) and 25 (D25) following orthotopic tumor inoculation. (**A**–**C**) Representative plots (**A**), frequency (**B**), and absolute number (**C**) of CD4^+^RANKL^+^ T cells. (**D**–**F**) Representative plots (**D**), frequency (**E**), and absolute number (**F**) of CD19^+^RANKL^+^ B cells. Data are presented as mean ± SD. Data were obtained from two independent experiments, with *n* = 5 mice per group, except where individual technical exclusions apply. Comparisons between naïve and EO771B-bearing mice at D11 and D25 were selected to evaluate early tumor-associated changes and their presence at the later stage of tumor progression. Statistical analyses were performed using unpaired two-tailed Student’s *t*-tests, with nominal *p* values reported without adjustment for multiple comparisons. Statistical significance is indicated as follows: * *p* < 0.05; ns, not significant.

We next investigated whether EO771B progression was associated with changes in RANKL-expressing populations within BM. Both the frequency and absolute number of RANKL-expressing CD4^+^ T lymphocytes increased significantly in EO771B-bearing mice compared with naïve controls at day 11, whereas no significant differences between groups were observed at day 25 (Figure 5A–C; Supplementary Table S3). Importantly, this early enrichment coincided with the trabecular bone alterations detected by Micro-CT and occurred within the early experimental window in which tumor-cell dissemination to BM was not detectable by clonogenic assay (Figure 2; Supplementary Figure S5; Supplementary Table S2). A similar temporal pattern was observed for RANKL-expressing CD8^+^ T lymphocytes. EO771B-bearing mice exhibited significant increases in both frequency and absolute number of RANKL-expressing CD8^+^ T cells at day 11, whereas these differences were no longer detected at day 25 (Supplementary Figure S9G–I).

B lymphocytes showed a similar pattern of RANKL expression. Increased frequencies and absolute numbers of RANKL-expressing CD19^+^ B cells were detected in BM at day 11, whereas no significant differences between naïve and EO771B-bearing mice were observed at day 25 (Figure 5D–F; Supplementary Table S3). Thus, the early stage of EO771B tumor progression was associated with enrichment of multiple RANKL-expressing adaptive immune populations within BM.

Collectively, these findings show that EO771B tumor progression is associated with early enrichment of RANKL-expressing CD4^+^ T, CD8^+^ T, and B lymphocytes within BM. This osteoimmune phenotype coincides temporally with early trabecular bone deterioration and precedes the experimental window in which tumor-cell dissemination to BM becomes detectable by clonogenic assay.

### 3.5. Adoptive Transfer of Tumor-Educated Leukocytes Promotes Trabecular Bone Deterioration in Recipient Mice

**Figure 6:**
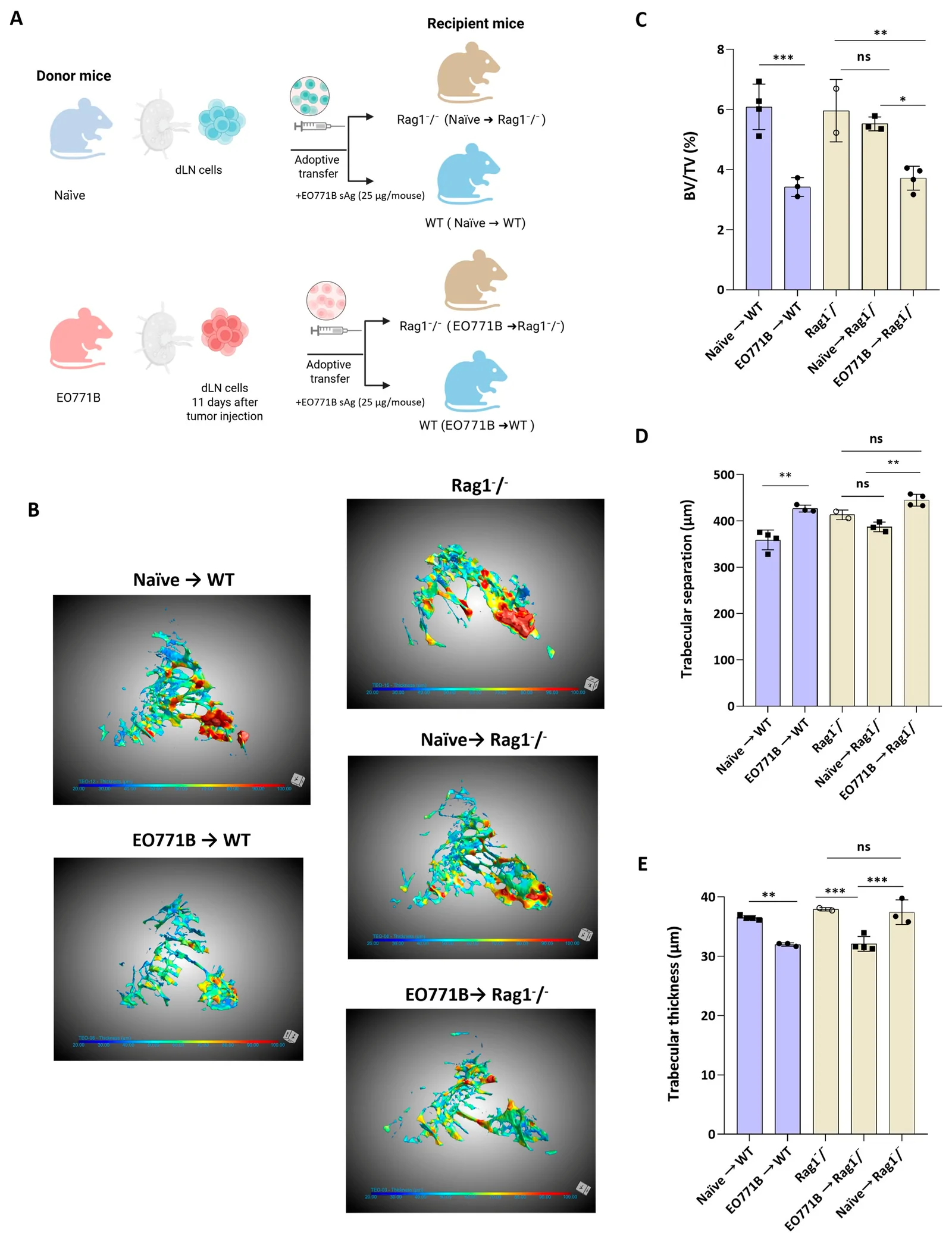
**Tumor-educated leukocytes induce trabecular bone deterioration following adoptive transfer.** (**A**) Schematic representation of the adoptive transfer experimental design. Total leukocytes were isolated from tumor-draining lymph nodes (dLNs) of naïve or EO771B-bearing wild-type (WT) C57BL/6 donor mice at day 11 after tumor inoculation and transferred into either immunocompetent WT or lymphopenic *Rag1*^−/−^ recipient mice. All transferred leukocyte preparations were co-administered with soluble EO771B tumor antigen (sAg; 25 μg/animal). The experimental groups comprised WT recipients receiving leukocytes from naïve donors (Naïve → WT), WT recipients receiving leukocytes from EO771B-bearing donors (EO771B → WT), *Rag1*^−/−^ recipients receiving leukocytes from naïve donors (Naïve → *Rag1*^−/−^), and *Rag1*^−/−^ recipients receiving leukocytes from EO771B-bearing donors (EO771B → *Rag1*^−/−^). An additional untreated *Rag1*^−/−^ group was included as a control. Recipient mice were euthanized 18 days after adoptive transfer for analysis of skeletal microarchitecture. (**B**) Representative three-dimensional micro-computed tomography (micro-CT) reconstructions of iliac trabecular bone from the indicated recipient groups. (**C**–**E**) Quantitative micro-CT analysis of trabecular bone parameters, including (**C**) bone volume fraction (BV/TV), (**D**) trabecular separation (**Tb.Sp**), and (**E**) trabecular thickness (Tb.Th). Data are presented as mean ± SD. Data were obtained from one independent experiment, with *n* = 4 for Naïve → WT, *n* = 3 for EO771B → WT, *n* = 2 for untreated *Rag1*^−/−^, *n* = 3 for Naïve → *Rag1*^−/−^, and *n* = 4 for EO771B → *Rag1*^−/−^. Statistical analyses were performed using one-way ANOVA followed by Tukey’s multiple comparisons test for each bone parameter. The indicated pairwise comparisons were selected according to the experimental objectives, and the reported *p* values were adjusted using Tukey’s procedure. Statistical significance is indicated as follows: * *p* < 0.05; ** *p* < 0.01; *** *p* < 0.001; ns, not significant.

To determine whether immune populations conditioned during EO771B progression can modulate skeletal homeostasis, total leukocytes isolated from dLNs of EO771B-bearing or tumor-free donors were adoptively transferred into lymphopenic Rag1-deficient (*Rag1*^−/−^) and immunocompetent C57BL/6 wild-type (WT) recipients (Figure 6A; Supplementary Table S4). Flow-cytometric analyses were subsequently performed to characterize T- and B-cell populations in the spleen and BM of recipient animals (Supplementary Figures S11 and S12). To investigate the effects of transferred leukocytes on trabecular bone microarchitecture, iliac bones were subjected to Micro-CT analysis 18 days following adoptive transfer (Figure 6B).

Recipients of leukocytes derived from EO771B-bearing donors exhibited alterations in trabecular microarchitecture compared with the corresponding control groups receiving leukocytes from naïve donors (Figure 6C–E; Supplementary Table S4). In both WT and *Rag1*^−/−^ recipients, transfer of EO771B-educated leukocytes resulted in reduced bone volume fraction (BV/TV) and trabecular thickness compared with the respective recipients of leukocytes from naïve donors (Figure 6C,E; Supplementary Table S4). Trabecular separation was increased in WT recipients of EO771B-educated leukocytes compared with WT recipients of naïve leukocytes, and a significant increase was also observed between the corresponding *Rag1*^−/−^ recipient groups (Figure 6D; Supplementary Table S4). Thus, despite their distinct immunological backgrounds, both recipient groups developed concordant alterations in BV/TV, trabecular thickness, and trabecular separation following transfer of tumor-educated leukocytes.

RANKL-expressing CD4^+^ T- and CD19^+^ B-cell populations were additionally evaluated in the spleen and BM of recipient mice at the experimental endpoint (Supplementary Figure S13). No significant differences in these RANKL-expressing populations were detected between recipients of leukocytes from naïve and EO771B-bearing donors, and RANKL-expressing cells were minimally detected or undetectable in BM (Supplementary Figure S13). Nevertheless, recipients of leukocytes from EO771B-bearing donors developed alterations in trabecular microarchitecture resembling those observed during EO771B tumor progression under the transfer protocol used. The effect observed in lymphopenic *Rag1*^−/−^ recipients is particularly informative because these animals lack endogenous mature T and B lymphocytes, demonstrating that the transferred leukocyte population can promote skeletal remodeling in this recipient background.

Collectively, these findings demonstrate that leukocytes isolated from tumor-dLNs during EO771B progression can promote trabecular bone deterioration following adoptive transfer, including in lymphopenic recipients. Together with the early enrichment of RANKL-expressing adaptive immune populations observed during EO771B progression, these findings support a functional contribution of tumor-educated immune cells to skeletal remodeling. However, the adoptive-transfer experiment does not establish a specific causal role for lymphocyte-derived RANKL in this effect.

## 4. Discussion

The present study demonstrates that progression of EO771B tumors is associated with expansion of RANKL-expressing lymphocyte populations, early trabecular bone deterioration, and subsequent detectable tumor-cell dissemination to the BM. Notably, skeletal alterations emerged before BM dissemination became detectable by the clonogenic assay used in this study and were partially reproduced following adoptive transfer of EO771B tumor-educated leukocytes, supporting a functional contribution of tumor-educated immune cells to skeletal remodeling. Together, these findings reinforce the concept that tumor progression and skeletal remodeling are dynamically interconnected processes rather than temporally independent events.

Previous observations from our group using metastatic 4T1 tumors demonstrated that expansion of functional tumor-specific CD4^+^RANKL^+^ T and CD19^+^RANKL^+^ B lymphocytes precedes detectable skeletal dissemination, promoting osteolytic alterations and favoring subsequent bone metastasis establishment [26,27]. Here, EO771B tumor progression recapitulated early osteoimmune alterations previously observed in the 4T1 model, including enrichment of RANKL-expressing lymphocyte populations and skeletal deterioration before BM dissemination became detectable. The observation of related osteoimmune phenotypes in two distinct syngeneic immunocompetent tumor–host systems, EO771B-C57BL/6 and 4T1-BALB/c, suggests that tumor-associated osteoimmune remodeling is not restricted to a single experimental model. Nevertheless, the present findings do not establish that the underlying mechanisms are identical across these systems.

The establishment of an EO771-derived metastatic breast cancer model in the C57BL/6 background provides an additional experimental platform for investigating the cellular and molecular mechanisms linking breast cancer progression, immune responses, and skeletal remodeling. The parental EO771 cell line has been characterized as a syngeneic mammary cancer model for C57BL/6 mice [30]; however, the EO771B population generated here was not molecularly recharacterized following serial in vivo selection, and therefore, no molecular subtype is assigned to the selected population in the present study. In conjunction with the 4T1-BALB/c system, the EO771B-C57BL/6 model enables comparative investigation across tumors with distinct biological characteristics and host genetic backgrounds, providing opportunities to dissect tumor-intrinsic and host-dependent contributions to osteoimmune remodeling and metastatic progression. Importantly, the C57BL/6 background further expands the experimental versatility of the EO771B model because of the broad availability of genetically modified and transgenic mouse strains on this background, facilitating future mechanistic interrogation of specific cellular populations and molecular pathways.

The interpretation of these findings gains additional complexity when considered alongside our observations in the non-metastatic 67NR setting [25,27]. Unlike metastatic tumors, 67NR progression induced increased bone mass associated with expansion of IFN-γ^+^IL-10^+^ CD8^+^ T cells and OPG-producing B lymphocytes despite the absence of detectable dissemination [25,27]. Collectively, these models suggest that primary mammary tumors can trigger systemic osteoimmune responses long before or even in the absence of metastatic establishment; however, the profile of skeletal remodeling appears to vary according to tumor-associated adaptive immune programs. Whereas metastatic tumors have been associated in our experimental models with skeletal deterioration and enrichment of RANKL-expressing populations, non-metastatic tumors may promote immune phenotypes associated with preservation or even gain of bone mass.

Changes in bone mass should therefore be interpreted cautiously, as skeletal competence depends not only on bone quantity but also on microarchitectural organization, turnover dynamics and biomechanical properties. This distinction is particularly relevant in breast cancer, in which skeletal metastases are predominantly osteolytic but may also display mixed osteolytic–osteoblastic characteristics, both contributing to skeletal fragility despite distinct effects on bone mass [1,30,31,32]. Likewise, bone mineral density (BMD) alone incompletely reflects bone quality, and higher BMD has paradoxically been associated with breast cancer incidence or heterogeneous clinical outcomes in some patient cohorts [33,34,35]. Thus, whether the increased bone mass observed in 67NR-bearing animals in our previous study [28] represents preservation of healthy skeletal homeostasis or an alternative form of tumor-induced remodeling remains uncertain.

A particularly relevant observation of the present study was the early enrichment of RANKL-expressing lymphocytes within BM. Both frequencies and absolute numbers of CD4^+^RANKL^+^, CD8^+^RANKL^+^ and CD19^+^RANKL^+^ populations increased at day 11, whereas significant differences relative to naïve controls were no longer detected at day 25. This temporal pattern is consistent with preferential involvement of these populations during early skeletal remodeling, although the two time points examined do not establish the complete kinetics of this response. Similar concepts have emerged from studies of pre-metastatic niche formation, in which inflammatory mediators, stromal alterations and immune-cell recruitment generate permissive microenvironments before arrival of disseminated tumor cells [36,37,38,39]. Rather than functioning solely as a passive metastatic target, bone marrow may represent an immunologically active compartment capable of responding dynamically to systemic tumor-derived cues. The temporal coincidence between early RANKL-expressing lymphocyte enrichment and trabecular bone deterioration supports an association between adaptive immune remodeling and the skeletal response observed in EO771B-bearing animals. However, the present experiments do not establish that lymphocyte-derived RANKL is responsible for the observed bone alterations. Importantly, such observations do not imply that lymphocyte-derived RANKL constitutes a dominant regulator of physiological bone remodeling but rather identify it as a candidate pathway for future mechanistic investigation in the context of tumor-associated skeletal remodeling. Emerging evidence further suggests that distinct forms of RANKL signaling may differentially contribute to physiological remodeling and pathological bone alterations, reinforcing the context-dependent nature of this pathway during metastatic progression [13].

EO771B progression was also associated with compartment-specific remodeling of RANKL-expressing lymphocytes within tumor-dLNs and the primary tumor microenvironment. Notably, these phenotypic changes occurred without corresponding expansion of total CD4^+^ or CD8^+^ T-cell populations in dLNs, indicating that changes in RANKL-expressing populations may occur independently of major alterations in the overall abundance of these T-cell subsets. The distinct behavior of CD4^+^RANKL^+^, CD8^+^RANKL^+^ and CD19^+^RANKL^+^ populations across lymphoid and tumor compartments further suggests that RANKL expression is dynamically regulated according to cellular and tissue context rather than simply reflecting changes in total lymphocyte abundance.

The adoptive transfer experiments provide additional functional support for the contribution of tumor-educated leukocytes to skeletal remodeling. Transfer of EO771B tumor-educated leukocytes reduced BV/TV and trabecular thickness in both WT and *Rag1*^−/−^ recipients, demonstrating that leukocytes isolated from tumor-dLNs can promote alterations in skeletal microarchitecture following adoptive transfer. The occurrence of this phenotype in *Rag1*^−/−^ recipients is particularly informative because these animals lack endogenous mature T and B lymphocytes, allowing the skeletal effects of the transferred leukocyte population to be evaluated in a lymphopenic background. Although RANKL-expressing lymphocyte populations were minimally detected or undetectable in BM at the experimental endpoint, the persistence of skeletal alterations may reflect earlier events following transfer that were not captured at the endpoint analyzed. Because soluble antigen was administered only at the time of transfer, without sustained antigenic stimulation throughout the experimental period, the phenotype of transferred cells may also have changed during the 18-day interval before analysis. Importantly, because total dLN leukocytes rather than purified RANKL-expressing populations were transferred, these experiments establish a functional effect of tumor-educated leukocytes but do not assign this effect to a specific lymphocyte subset or demonstrate that RANKL is required for the observed skeletal alterations. Future experiments using cell-subset-specific approaches or RANKL blockade will be required to establish this mechanistic relationship.

The present findings may help contextualize apparently heterogeneous clinical observations reported over the last decade regarding bone homeostasis in patients with breast cancer [4,5,6,7,8,9,10]. Several studies have described reduced BMD, increased prevalence of osteopenia/osteoporosis and elevated fracture risk even in newly diagnosed patients or individuals younger than conventional screening thresholds, frequently before prolonged endocrine therapy exposure or overt skeletal metastasis [4,5,6,7,8,9]. Conversely, other reports have associated lower BMD with reduced recurrence risk or improved prognosis in specific patient populations, suggesting more complex relationships between bone remodeling and tumor progression than previously appreciated [10].

Our experimental findings raise the possibility that these apparently discordant clinical patterns may not exclusively reflect demographic, hormonal or treatment-associated differences, but could also encompass distinct skeletal responses associated with tumor biology. In this context, the contrasting phenotypes observed in metastatic 4T1/EO771B and non-metastatic 67NR models provide an experimental framework in which biologically divergent tumors are associated with distinct osteoimmune trajectories. Although direct translation to patients remains to be established, this framework raises the possibility that skeletal parameters may capture, at least in part, systemic tumor-immune interactions occurring during disease progression rather than simply the consequences of aging, endocrine status or treatment toxicity. Consistent with this interpretation, our previous work demonstrated that RANKL-expressing T-cell and OPG-producing B-cell immune signatures in human breast cancer were associated with subsequent bone-metastatic outcomes, with RANKL-expressing T-cell populations associated with increased risk of bone metastasis and OPG-producing B-cell populations with reduced risk [25]. These clinical observations support the translational relevance of the osteoimmune phenotypes investigated here, while not establishing a causal role for these populations in human skeletal metastasis.

Several limitations should be considered when interpreting the present findings. BM tumor-cell dissemination was assessed using a clonogenic assay, which detects cells capable of surviving and forming colonies under the culture conditions employed but does not constitute histological demonstration of established bone metastases. Accordingly, the absence of colonies at the early experimental time point should be interpreted as absence of detectable clonogenic tumor-cell dissemination rather than absolute absence of tumor cells. In addition, Micro-CT identifies alterations in trabecular architecture but does not directly establish the cellular mechanism responsible for bone loss. Finally, although the adoptive-transfer experiments demonstrate a functional effect of tumor-educated leukocytes on skeletal microarchitecture, the use of total dLN leukocytes and the absence of RANKL-specific blockade prevent attribution of this effect to a particular lymphocyte population or to RANKL itself. These questions can now be addressed using the EO771B-C57BL/6 system in combination with cell-specific genetic or pharmacological approaches.

## 5. Conclusions

In conclusion, our findings demonstrate that EO771B tumor progression is associated with distal osteoimmune remodeling before tumor-cell dissemination to BM becomes detectable by clonogenic assay, characterized by early enrichment of RANKL-expressing adaptive immune populations and deterioration of trabecular architecture. The observation of related early skeletal alterations in distinct syngeneic breast cancer models, together with the functional effects of tumor-educated leukocytes on bone microarchitecture, supports tumor-associated immune remodeling as a biologically relevant component of the skeletal response to breast cancer progression. The EO771B-C57BL/6 model therefore provides an additional experimental platform for mechanistic investigation of the cellular and molecular interactions linking breast tumors, adaptive immunity, and the skeletal microenvironment.

## Data Availability

The underlying numerical data supporting the findings of this study and additional experimental data are available from the corresponding author upon reasonable request.

## Supplementary Materials

The following supporting information can be downloaded at: https://www.mdpi.com/article/10.3390/biology15212014/s1, Figure S1. Generation of the EO771-derived selected breast cancer population EO771B. Schematic representation of the serial multi-site in vivo selection strategy used to generate EO771B. Parental EO771 cells (2.5 × 10^5^ cells per inoculation route) were inoculated into female C57BL/6 mice through orthotopic (fourth mammary fat pad), intravenous (retro-orbital plexus), and intratibial routes. Animals were anesthetized by intraperitoneal administration of ketamine (90 mg/kg) and xylazine (10 mg/kg) before the corresponding experimental procedures. Twenty-five days after inoculation, cells were recovered from primary tumors, dLNs, lungs, liver, spleen and tibia-derived bone marrow (BM), independently expanded in vitro, pooled, and reinoculated into new recipient mice using the same inoculation routes and cell number (2.5 × 10^5^ cells per route). This procedure was repeated for three consecutive cycles. After completion of the third cycle, pooled cells (5 × 10^5^ cells) were inoculated exclusively through the orthotopic route, and spontaneous tumor-cell dissemination was assessed 35 days later. The resulting selected EO771-derived population was designated EO771B. Figure S2. Representative gating strategies used for immunophenotypic characterization of lymphocyte populations in dLNs and the primary tumor microenvironment. (A) Representative flow cytometry plots illustrating the sequential gating strategy employed to identify lymphocyte populations in (A) tumor-dLNs, (B) primary tumor microenvironment (TME) from EO771B-bearing mice. Cell populations were initially selected based on forward and side scatter parameters (FSC-A versus SSC-A) to exclude debris, followed by singlet discrimination using FSC-A versus FSC-H. Viable cells were subsequently identified through exclusion of Live/Dead-positive events. Representative plots illustrate the gating hierarchy applied throughout the flow cytometric analyses. Figure S3. Representative gating strategy used for immunophenotypic characterization of lymphocyte populations in bone marrow. Representative flow cytometry plots illustrating the sequential gating strategy employed to identify lymphocyte populations in the bone marrow (BM) from EO771B-bearing mice. Cell populations were initially selected based on forward and side scatter parameters (FSC-A versus SSC-A) to exclude debris, followed by singlet discrimination using FSC-A versus FSC-H. Viable cells were subsequently identified through exclusion of Live/Dead-positive events. Representative plots illustrate the gating hierarchy applied throughout the flow cytometric analyses. Figure S4. Additional characterization of mice bearing parental EO771 or EO771B tumors following orthotopic inoculation. (A) Primary tumor weight and (B) spleen weight in mice bearing parental EO771 or EO771B tumors. Total cellularity of (C) tumor-draining lymph nodes (dLNs) and (D) bone marrow (BM) from parental EO771- and EO771B-bearing mice. (E) Incidence of detectable clonogenic tumor-cell dissemination to dLNs and BM at day 35 after orthotopic inoculation. Quantitative data are presented as mean ± SD. Data were obtained from two independent experiments (*n* = 6 mice per group). Statistical comparisons of quantitative outcomes were performed using unpaired two-tailed Student’s *t*-tests, where applicable. Nominal *p* values are reported without adjustment for multiple comparisons. Dissemination incidence is expressed as the number of animals with detectable clonogenic colonies relative to the total number of animals evaluated. Statistical significance is indicated as follows: ** *p* < 0.01; ns, not significant. Figure S5. Clonogenic assessment of tumor-cell dissemination during EO771B tumor progression. (A) Clonogenic tumor-cell burden in tumor-draining lymph nodes (dLNs) and (B) bone marrow (BM) at days 10 (D10) and 30 (D30) after orthotopic inoculation of EO771B cells. Representative images of clonogenic cultures are shown above the corresponding quantitative analyses. Colony numbers are expressed relative to the number of cells plated, as indicated on the respective y-axes. Data are representative of three independent experiments, with *n* = 6 mice per group. Statistical comparisons between D10 and D30 were performed using unpaired two-tailed Student’s *t*-tests. Nominal *p* values are reported without adjustment for multiple comparisons. Statistical significance is indicated as *p* < 0.05 (*). Figure S6. EO771B progression differentially modulates total T- and B-lymphocyte populations in tumor-draining lymph nodes. Representative flow cytometry plots and quantification of total CD3^+^ T, CD4^+^ T, CD8^+^ T, and CD19^+^ B lymphocytes in tumor-draining lymph nodes (dLNs) from naïve and EO771B-bearing mice at days 11 (D11) and 25 (D25) following orthotopic EO771B inoculation. (A–C) Representative plots (A), frequency (B), and absolute number (C) of CD3^+^ T cells. (D–H) Representative plots (D) and quantification of the frequency and absolute number of CD4^+^ (E–F) and CD8^+^ (G–H) T cells within the CD3^+^ compartment. (I–K) Representative plots (I), frequency (J), and absolute number (K) of CD19^+^ B cells. Data are presented as mean ± SD. Data were obtained from two independent experiments, with *n* = 5 mice per group. Comparisons between naïve and EO771B-bearing mice at each experimental time point were performed using unpaired two-tailed Student’s *t*-tests. Nominal *p* values are reported without adjustment for multiple comparisons. Statistical significance is indicated as follows: ** *p* < 0.01; *** *p* < 0.001; **** *p* < 0.0001; ns, not significant. Figure S7. Lymphocyte composition of the primary tumor microenvironment changes during EO771B progression. Representative flow cytometry plots and quantification of total CD3^+^ T, CD4^+^ T, CD8^+^ T, and CD19^+^ B lymphocytes within the primary tumor microenvironment (TME) at days 11 (D11) and 25 (D25) following orthotopic EO771B inoculation. (A–C) Representative plots (A), frequency (B), and absolute number (C) of CD3^+^ T cells. (D–H) Representative plots (D) and quantification of the frequency and absolute number of CD4^+^ (E,F) and CD8^+^ (G,H) T cells within the CD3^+^ compartment. (I–K) Representative plots (I), frequency (J), and absolute number (K) of CD19^+^ B cells. Data are presented as mean ± SD. Data were obtained from 2 independent experiments, with *n* = 5 mice per group. Statistical analyses were performed using unpaired two-tailed Student’s *t*-test. Statistical significance is indicated as follows: * *p* < 0.05; ** *p* < 0.01; ns, not significant. Figure S8. Regulatory T-cell populations in tumor-dLNs and the primary tumor microenvironment during EO771B progression. Representative flow cytometry plots and quantification of CD4^+^Foxp3^+^ regulatory T cells in tumor-dLNs and the primary tumor microenvironment (TME) at days 11 (D11) and 25 (D25) following orthotopic EO771B inoculation. (A–C) Representative flow cytometry plots (A), frequency (B), and absolute number (C) of CD4^+^Foxp3^+^ T cells in dLNs from naïve and EO771B-bearing mice at D11 and D25. Frequencies are expressed as the percentage of Foxp3^+^ cells within the CD4^+^ T-cell population. (D–F) Representative flow cytometry plots (D), frequency (E), and absolute number (F) of CD4^+^Foxp3^+^ T cells within the primary TME at D11 and D25. Data are presented as mean ± SD. Data were obtained from two independent experiments, with *n* = 5 mice per group. Comparisons between naïve and EO771B-bearing mice at each time point in dLNs, and between D11 and D25 in the primary TME, were performed using unpaired two-tailed Student’s *t*-tests. Nominal *p* values are reported without adjustment for multiple comparisons. Statistical significance is indicated as follows: * *p* < 0.05; ** *p* < 0.01; ns, not significant. Figure S9. RANKL expression by CD8^+^ T cells. Representative flow cytometry plots and quantification of RANKL expression by CD8^+^ T lymphocytes in tumor-dLNs, the primary tumor microenvironment (TME), and bone marrow (BM) at days 11 (D11) and 25 (D25) following orthotopic EO771B inoculation. (A–C) Representative plots (A), frequency (B), and absolute number (C) of CD8^+^RANKL^+^ T cells in dLNs from naïve and EO771B-bearing mice. (D–F) Representative plots, including fluorescence-minus-one (FMO) control (D), frequency (E), and absolute number (F) of CD8^+^RANKL^+^ T cells within the primary TME at D11 and D25. (G–I) Representative plots (G), frequency (H), and absolute number (I) of CD8^+^RANKL^+^ T cells in BM from naïve and EO771B-bearing mice. Data are presented as mean ± SD. Data were obtained from two independent experiments, with *n* = 4–5 mice per group. Comparisons between naïve and EO771B-bearing mice at each experimental time point in dLNs and BM, and between D11 and D25 in the primary TME, were performed using unpaired two-tailed Student’s *t*-tests. Nominal *p* values are reported without adjustment for multiple comparisons. Statistical significance is indicated as follows: * *p* < 0.05; ns, not significant. Figure S10. Lymphocyte composition of bone marrow. Representative flow cytometry plots and quantification of total CD3^+^ T, CD4^+^ T, CD8^+^ T, and CD19^+^ B lymphocytes within BM from naïve and EO771B-bearing mice at days 11 (D11) and 25 (D25) following orthotopic EO771B inoculation. (A–C) Representative plots (A), frequency (B), and absolute number (C) of CD3^+^ T cells. (D–H) Representative plots (D) and quantification of the frequency and absolute number of CD4^+^ (E,F) and CD8^+^ (G,H) T cells within the CD3^+^ compartment. (I–K) Representative plots (I), frequency (J), and absolute number (K) of CD19^+^ B cells. Data were obtained from two independent experiments, with *n* = 5 mice per group. Comparisons between naïve and EO771B-bearing mice at each experimental time point were performed using unpaired two-tailed Student’s *t*-tests. Nominal *p* values are reported without adjustment for multiple comparisons. Statistical significance is indicated as follows: * *p* < 0.05; ** *p* < 0.01; *** *p* < 0.001; **** *p* < 0.0001; ns, not significant. Figure S11. T- and B-cell populations following adoptive transfer of leukocytes from naïve or EO771B-bearing mice. (A) Representative flow cytometry plots of CD3 T and CD19 B cells from spleen and bone marrow (BM) of wild-type (WT) or *Rag1*^−/−^ recipient mice following adoptive transfer of total leukocytes isolated from dLNs of naïve or EO771B-bearing WT donors. (B–E) Frequency (B) and absolute number (C) of CD3^+^ T cells and frequency (D) and absolute number (E) of CD19^+^ B cells in the spleen. (F–I) Frequency (F) and absolute number (G) of CD3^+^ T cells and frequency (H) and absolute number (I) of CD19^+^ B cells in BM. Data are presented as mean ± SD. Data were obtained from one independent experiment, with *n* = 3–4 mice per group. Statistical analyses were performed using one-way ANOVA followed by Tukey’s multiple comparisons test for each outcome. The indicated comparisons included Naïve → WT versus EO771B → WT and Naïve → *Rag1*^−/−^ versus EO771B → *Rag1*^−/−^. Reported *p* values were adjusted for multiple comparisons using Tukey’s procedure. Statistical significance is indicated as ns, not significant. Figure S12. CD4^+^ and CD8^+^ T-cell populations following adoptive transfer of leukocytes from naïve or EO771B-bearing mice. (A) Representative flow cytometry plots of CD4^+^ T and CD8^+^ T cells from spleen and bone marrow (BM) of wild-type (WT) or *Rag1*^−/−^ recipient mice following adoptive transfer of total leukocytes isolated from dLNs of naïve or EO771B-bearing WT donors. (B–E) Frequency (B) and absolute number (C) of CD4^+^ T cells and frequency (D) and absolute number (E) of CD8^+^ T cells in the spleen. (F–I) Frequency (F) and absolute number (G) of CD4^+^ T cells and frequency (H) and absolute number (I) of CD8^+^ T cells in the bone marrow. Data are presented as mean ± SD. Data were obtained from one independent experiment, with *n* = 3–4 mice per group. Statistical analyses were performed using one-way ANOVA followed by Tukey’s multiple comparisons test for each outcome. The indicated comparisons included Naïve → WT versus EO771B → WT and Naïve → *Rag1*^−/−^ versus EO771B → *Rag1*^−/−^. Reported *p* values were adjusted for multiple comparisons using Tukey’s procedure. Statistical significance is indicated as ns, not significant. Figure S13. RANKL expression by T and B cells following adoptive transfer of leukocytes from naïve or EO771B-bearing mice. (A,F) Representative flow cytometry plots of RANKL-expressing CD4^+^ T cells (A) and CD19^+^ B cells (F) from spleen and bone marrow (BM) of wild-type (WT) or *Rag1*^−/−^ recipient mice following adoptive transfer of total leukocytes isolated from dLNs of naïve or EO771B-bearing WT donors. (B,C) Frequency (B) and absolute number (C) of CD4^+^RANKL^+^ T cells in the spleen. (D,E) Frequency (D) and absolute number (E) of CD4^+^RANKL^+^ T cells in the iliac bone marrow. (F,G) Frequency (F) and absolute number (G) of CD19^+^RANKL^+^ B cells in the spleen, and frequency (H) and absolute number (I) of CD19^+^RANKL^+^ B cells in the BM. Data are presented as mean ± SD. Data were obtained from one independent experiment, with *n* = 3–4 mice per group. Statistical analyses were performed using one-way ANOVA followed by Tukey’s multiple comparisons test for each outcome. The indicated comparisons included Naïve → WT versus EO771B → WT and Naïve → *Rag1*^−/−^ versus EO771B → *Rag1*^−/−^. Reported *p* values were adjusted for multiple comparisons using Tukey’s procedure. Statistical significance is indicated as ns, not significant. Table S1: Antibodies used for flow cytometry analyses. The table lists the antibodies used for immunophenotypic characterization of murine cell populations, including their target antigens, species reactivity, clones, manufacturers, fluorochrome conjugates, and working dilutions. Anti-CD16/CD32 antibody was used for Fc receptor blocking prior to immunostaining. BD, BD Biosciences; PE, phycoerythrin; FITC, fluorescein isothiocyanate; APC, allophycocyanin; BV421, Brilliant Violet 421; RANKL, receptor activator of nuclear factor κB ligand; Table S2: Statistical analysis of trabecular bone microarchitecture during EO771B breast cancer progression. Trabecular bone microarchitecture was evaluated by micro-computed tomography (MicroCT) of iliac bones collected from tumor-free (Naïve) and EO771B-bearing C57BL/6 mice at days 11 (D11) and 25 (D25) following orthotopic tumor inoculation. The evaluated parameters included bone volume (BV), bone volume fraction (BV/TV), bone surface area (BS), trabecular separation (Tb.Sp), trabecular thickness (Tb.Th), and trabecular number (Tb.N), corresponding to Figure 2B–G. Statistical comparisons were selected according to the experimental objectives. Naïve mice were compared with EO771B-bearing mice at D11 and D25 to investigate tumor-associated alterations in skeletal microarchitecture at early and later stages of tumor progression, respectively. Comparisons between D11 and D25 were performed to evaluate additional changes in bone microarchitecture during tumor progression. Statistical analyses were performed using two-sided unpaired Student’s *t*-tests. All reported *p* values are nominal and were not adjusted for multiple comparisons. Data were obtained from two independent experiments, with *n* = 5 mice per group. The Naïve group comprised tumor-free control mice from the D11 and D25 experimental series, pooled to provide a baseline reference group. Statistical significance is indicated as follows: * *p* < 0.05; ** *p* < 0.01; *** *p* < 0.001; ns, not significant; Table S3: Statistical comparisons were selected according to the original experimental objectives, focusing on tumor-associated changes in RANKL-expressing CD4^+^ T and CD19^+^ B lymphocyte populations at early (D11) and later (D25) stages of EO771B tumor progression. In tumor-draining lymph nodes (dLNs) and bone marrow (BM), naïve mice were compared with EO771B-bearing mice at each experimental time point. Within the primary tumor microenvironment (TME), comparisons were performed between independent groups of EO771B-bearing mice at D11 and D25. Both relative frequencies and absolute cell numbers were evaluated as complementary outcomes. Statistical analyses were performed using two-sided unpaired Student’s *t*-tests. All *p* values are nominal (unadjusted for multiple comparisons). Statistical significance is indicated as follows: * *p* < 0.05; ** *p* < 0.01; *** *p* < 0.001; ns, not significant; Table S4: One-way ANOVA followed by Tukey’s multiple comparisons test for trabecular bone parameters following adoptive transfer of EO771B tumor-educated leukocytes. Trabecular bone microarchitecture was evaluated by micro-computed tomography (MicroCT) 18 days after adoptive transfer of total leukocytes isolated from tumor-draining lymph nodes (dLNs) of naïve or EO771B-bearing C57BL/6 donor mice. Leukocytes were transferred into wild-type (WT) or *Rag1*^−/−^ recipient mice. An additional group of untreated *Rag1*^−/−^ mice was included as a control. Bone volume fraction (BV/TV), trabecular thickness (Tb.Th), and trabecular separation (Tb.Sp) were analyzed separately using one-way ANOVA followed by Tukey’s multiple comparisons test. The table presents the Tukey-adjusted *p* values for the biologically relevant pairwise comparisons shown in Figure 6. The analysis included five experimental groups: Naïve → WT (*n* = 4), EO771B → WT (*n* = 3), untreated *Rag1*^−/−^ (*n* = 2), Naïve → *Rag1*^−/−^ (*n* = 3), and EO771B → *Rag1*^−/−^ (*n* = 4). Data were obtained from a single independent experiment. Statistical significance is indicated as follows: * *p* < 0.05; ** *p* < 0.01; *** *p* < 0.001; ns, not significant. All reported *p* values were adjusted for multiple comparisons using Tukey’s procedure.
