# Supplementary material for: An EO771-Derived Metastatic Breast Cancer Model Exhibits RANKL-Associated Osteoimmune Remodeling Before Detectable Bone-Marrow Dissemination
Source: Biology (Basel). 2026 Nov 5;15(21):2014. doi: 10.3390/biology15212014 (PMC13761562; doi:10.3390/biology15212014)
Supplement: Supplementary file 1 [file biology-15-02014-s001.zip › biology-4596185-supplementary.pdf]

**Supplementary Figure S1**

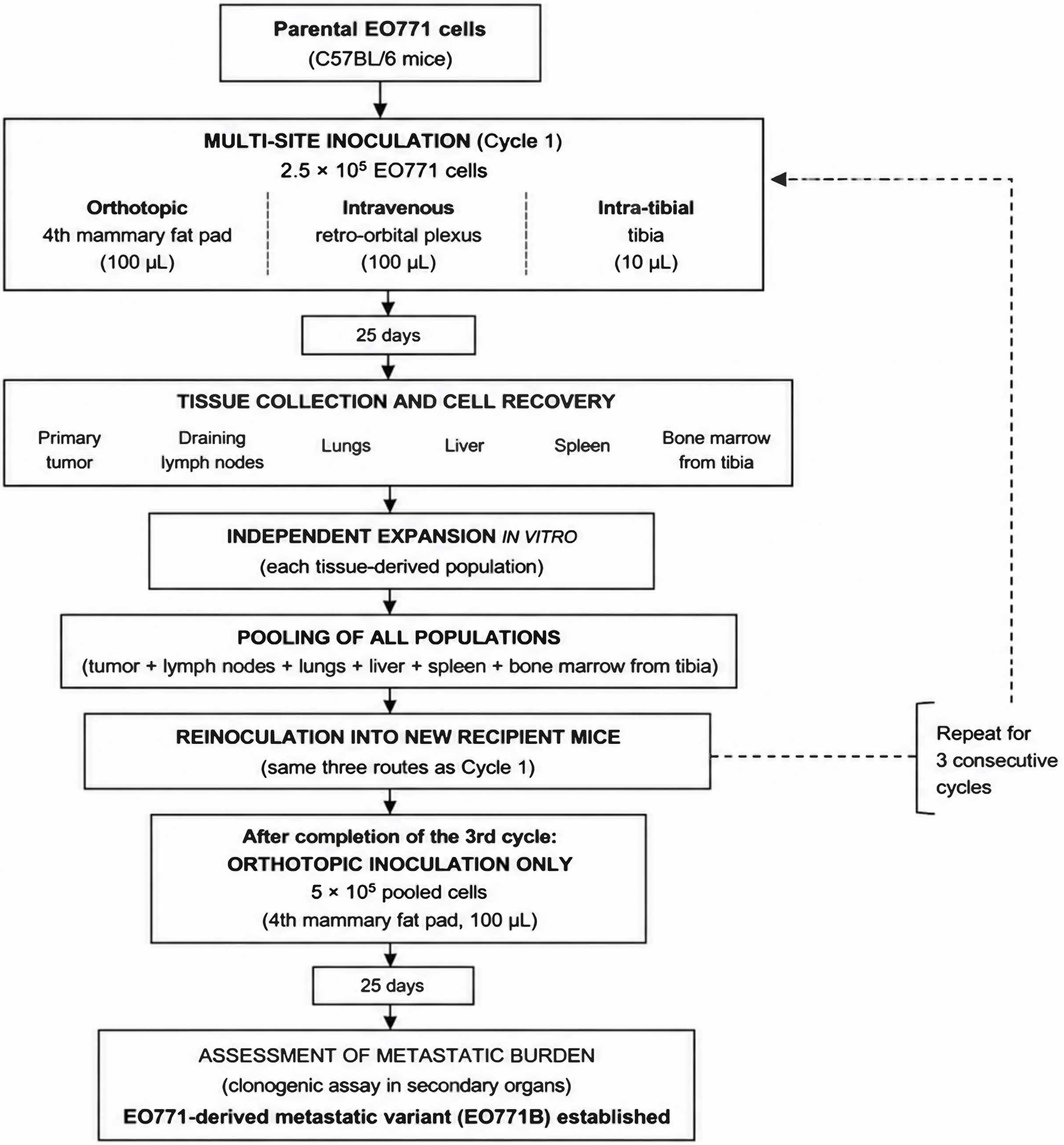

## Supplementary Figure S2

### Gating strategy

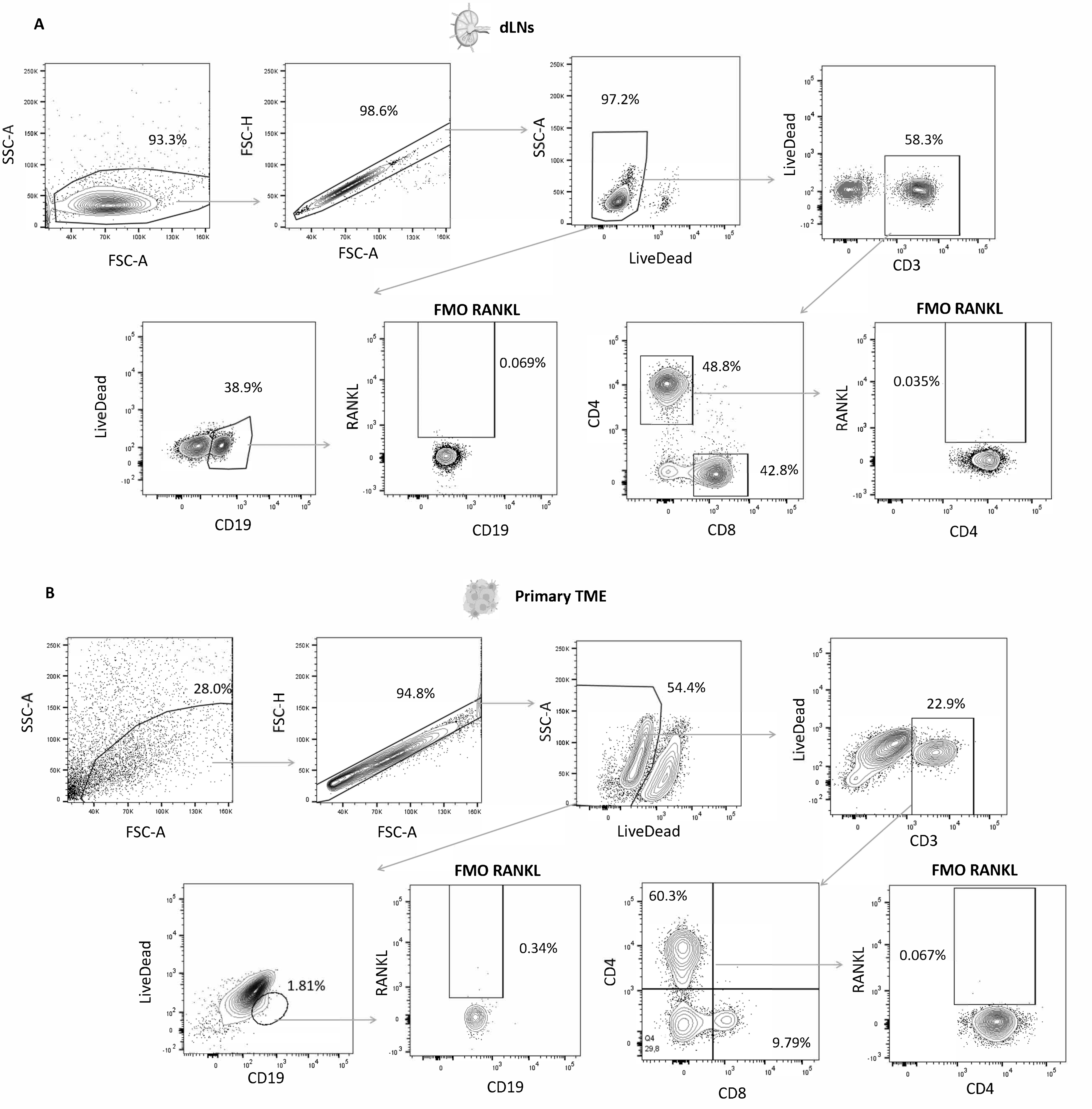

**Supplementary Figure S3**

## Gating strategy

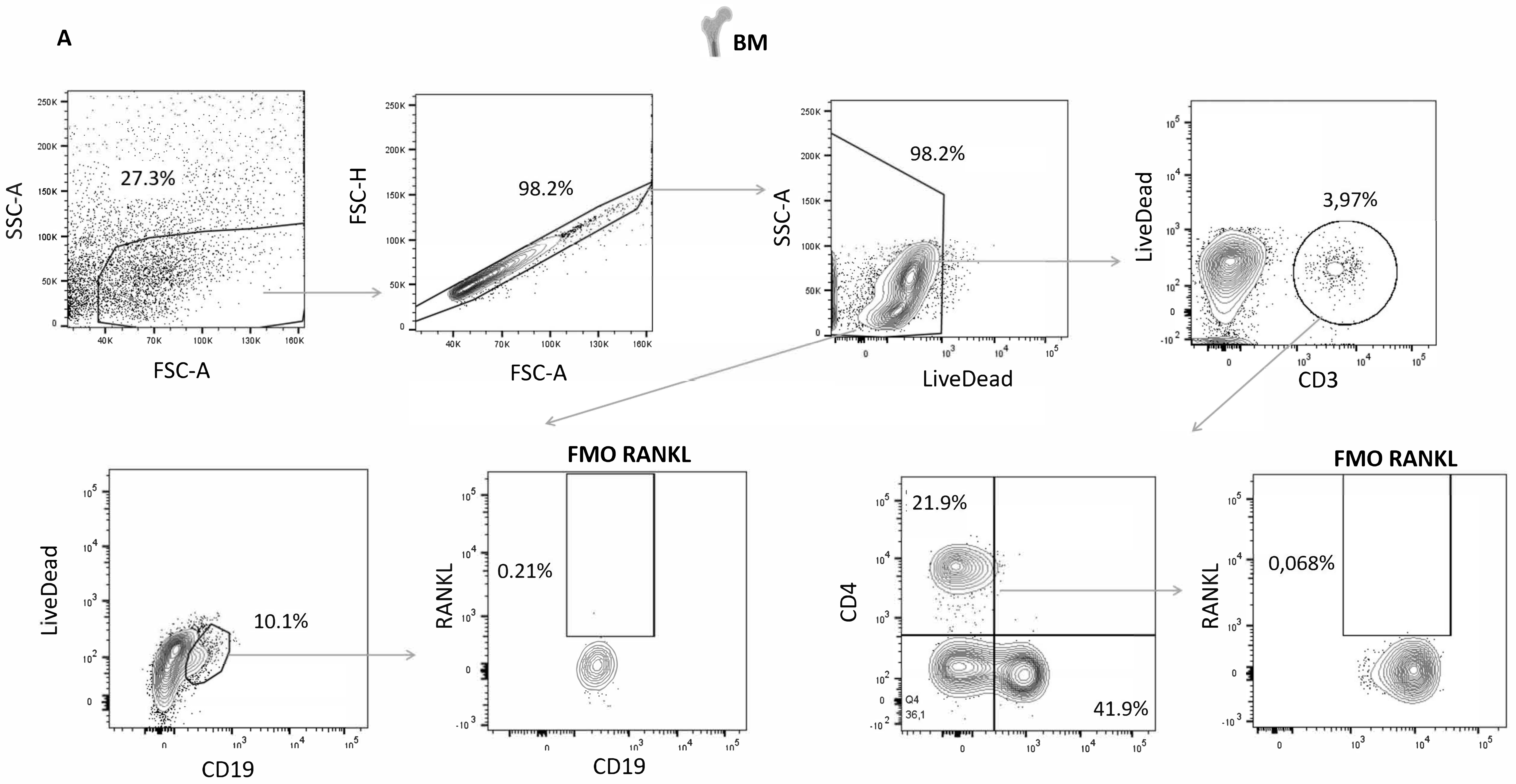

**Supplementary Figure S4**

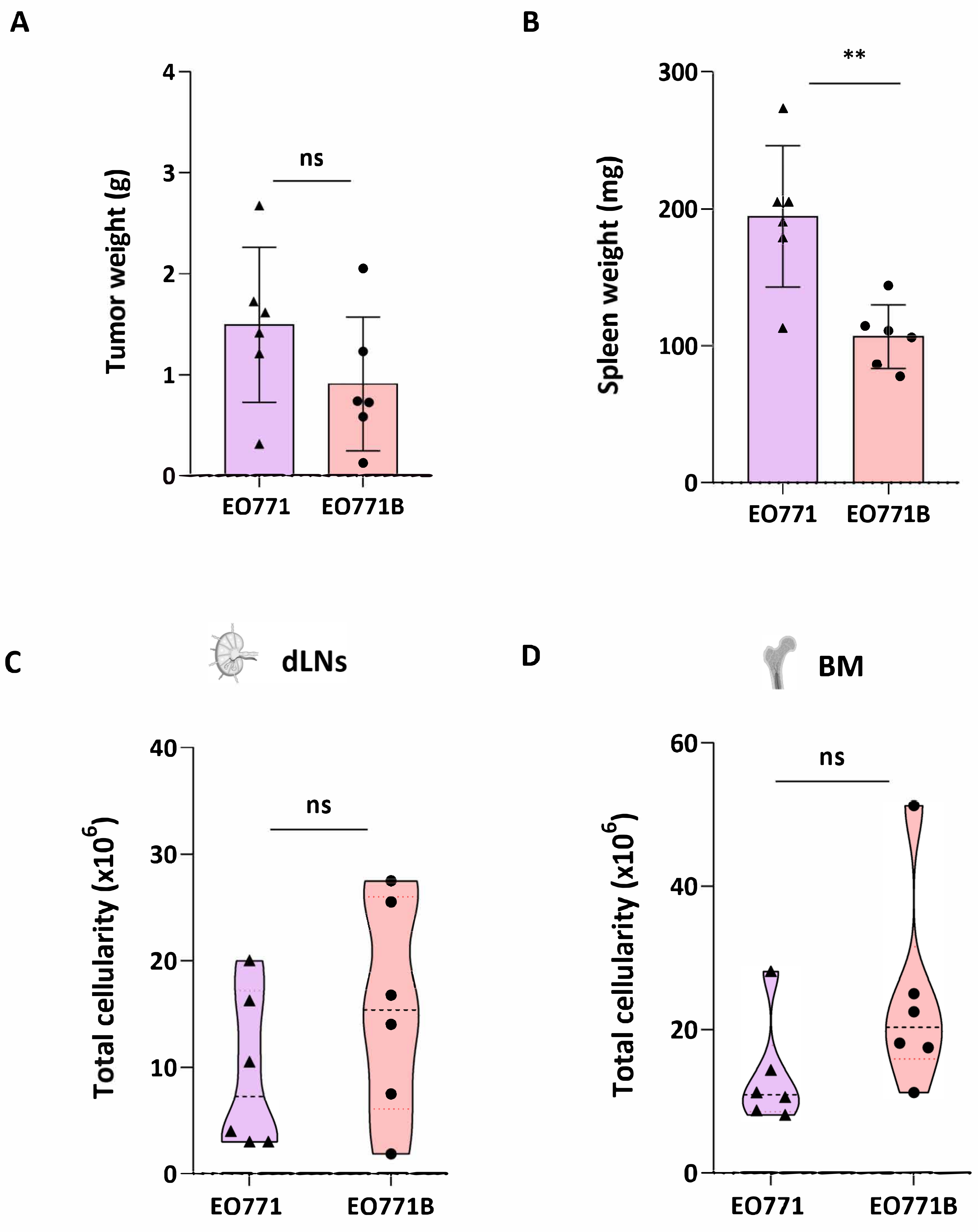


E

**Incidence of Clonogenic colonies at D35**

| Lineage / Organ | dLNs | BM |
|---|---|---|
| **EO771** | 60% | 0% |
| **EO771B** | 80% | 100% |

Supplementary Figure S5

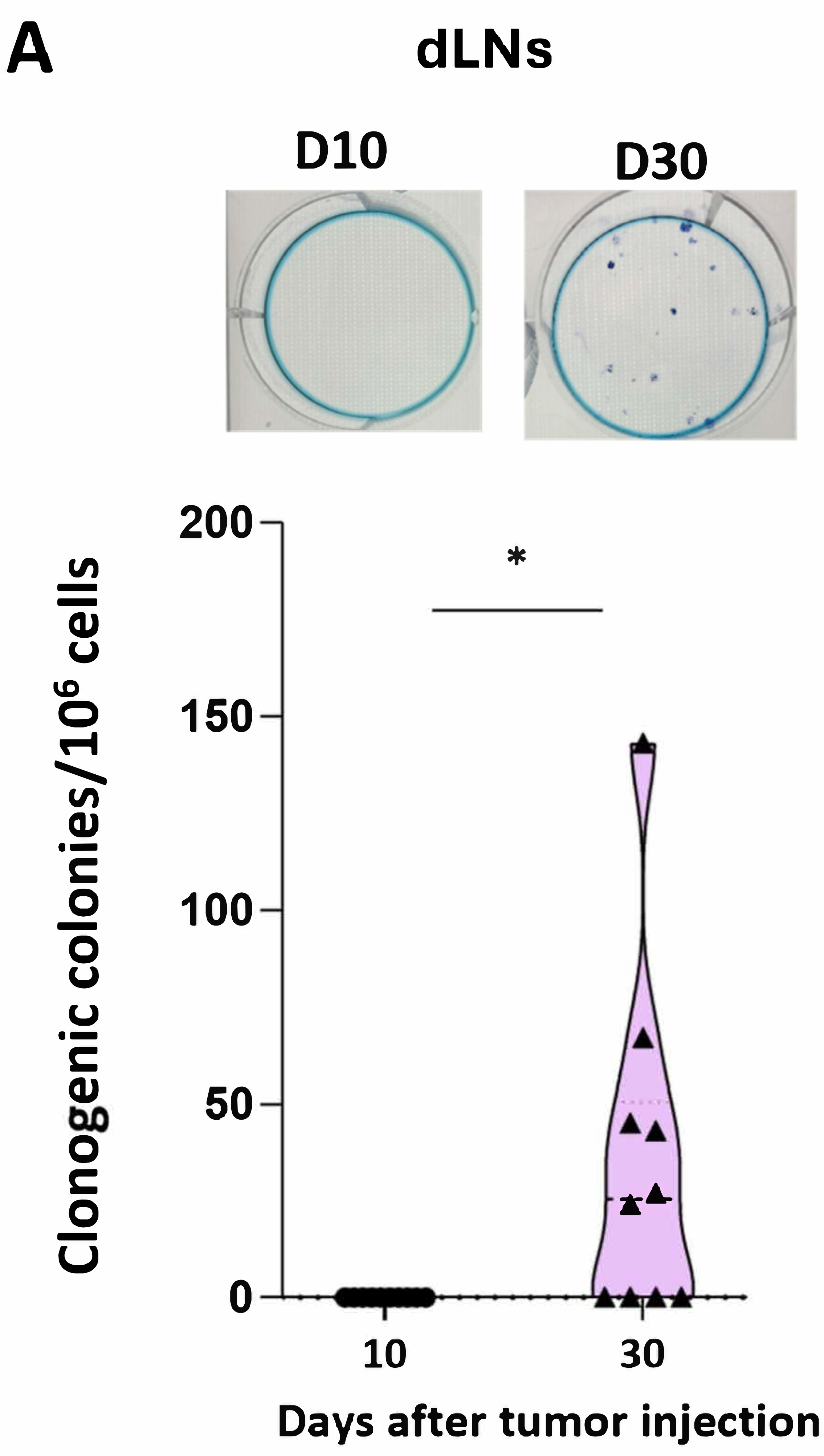


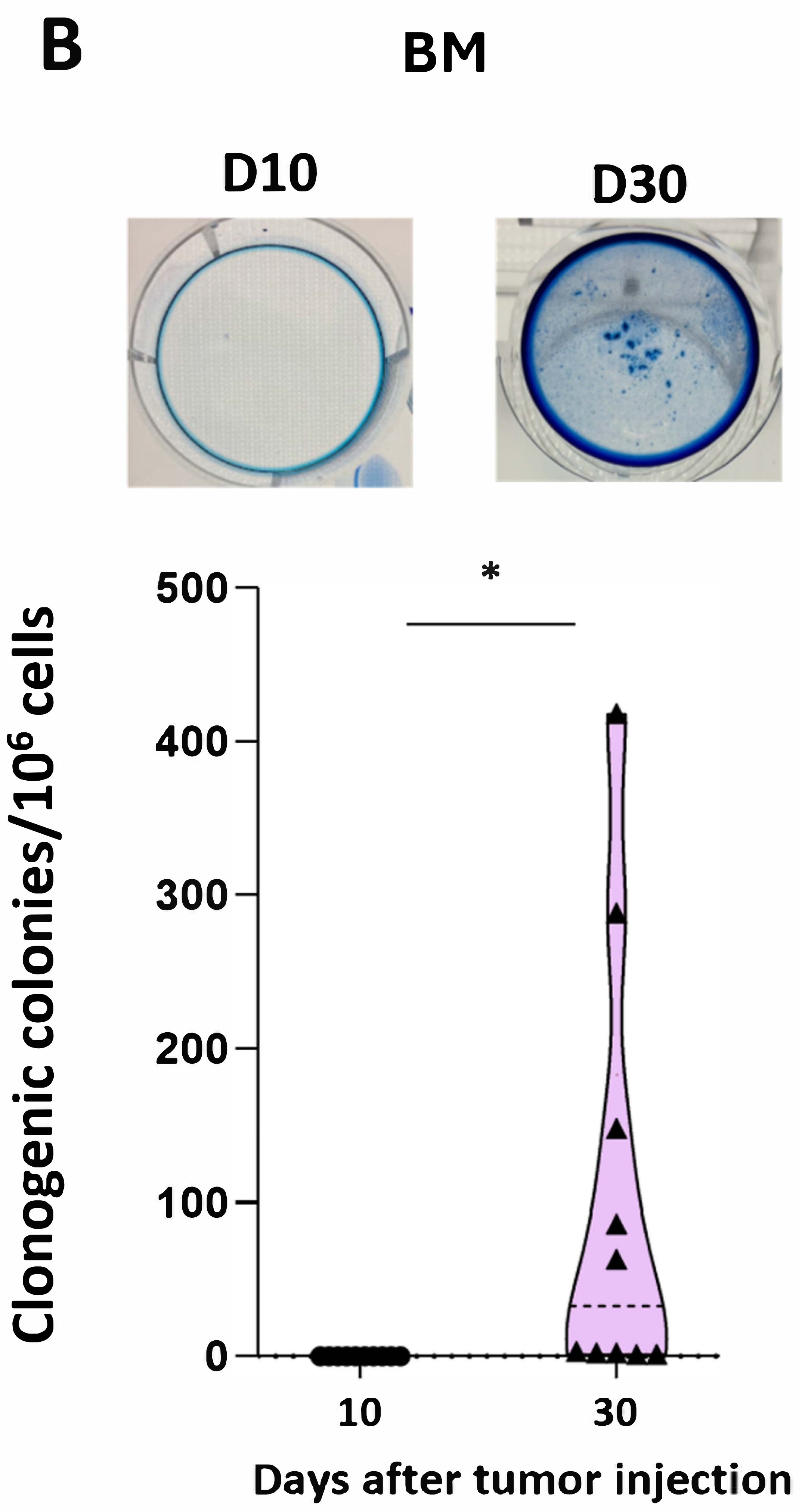

## Supplementary Figure S6

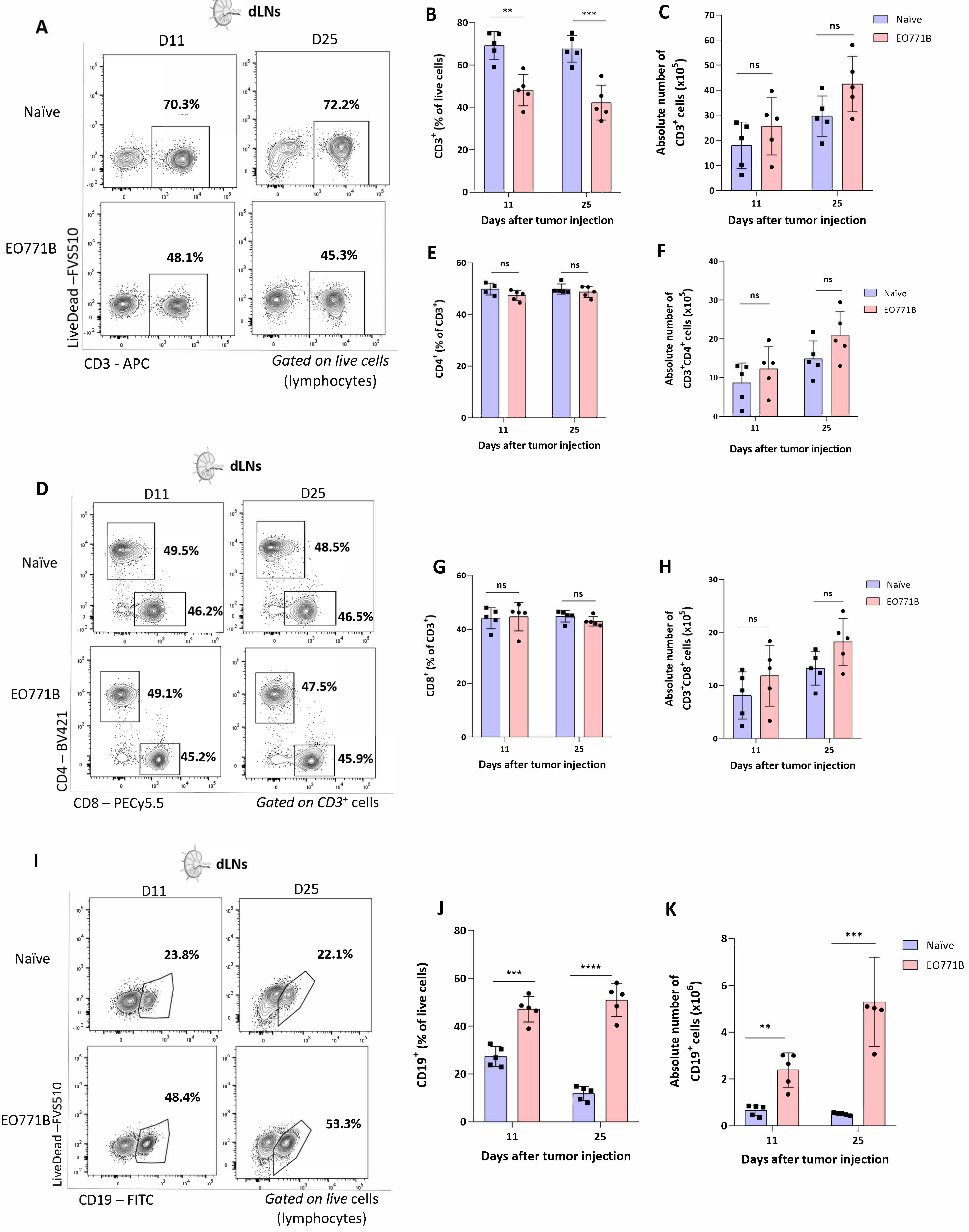

## Supplementary Figure S7

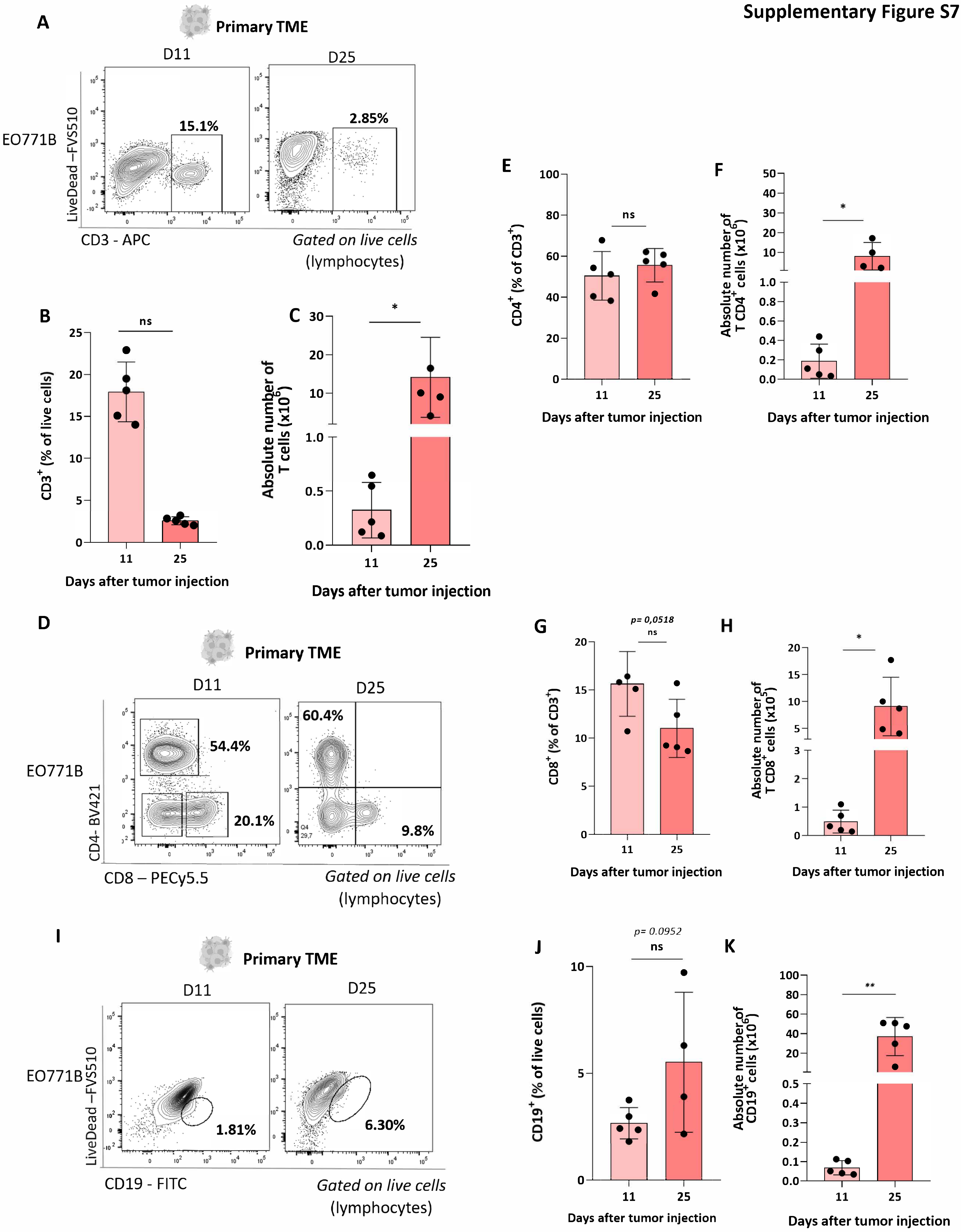

**Supplementary Figure S8**

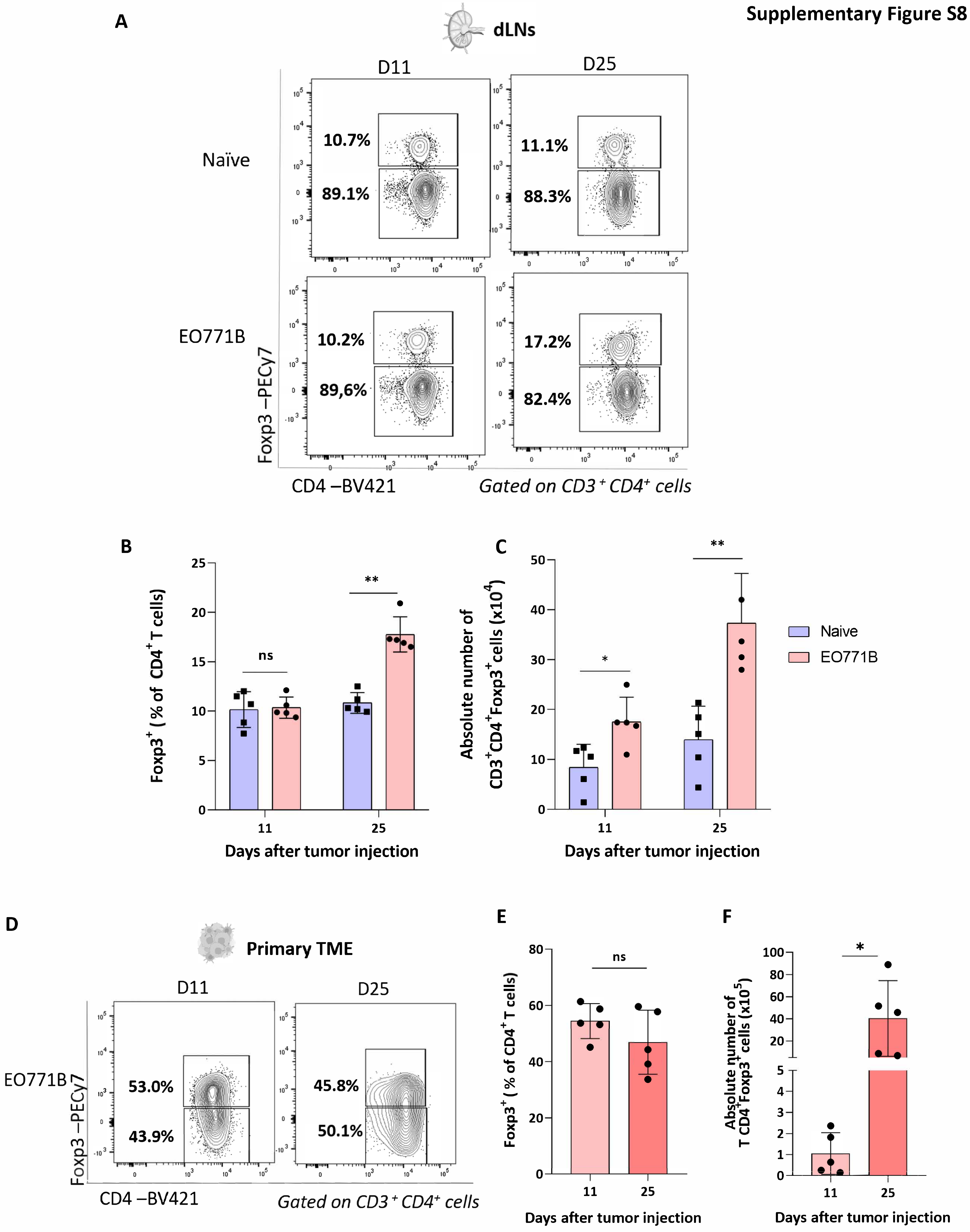

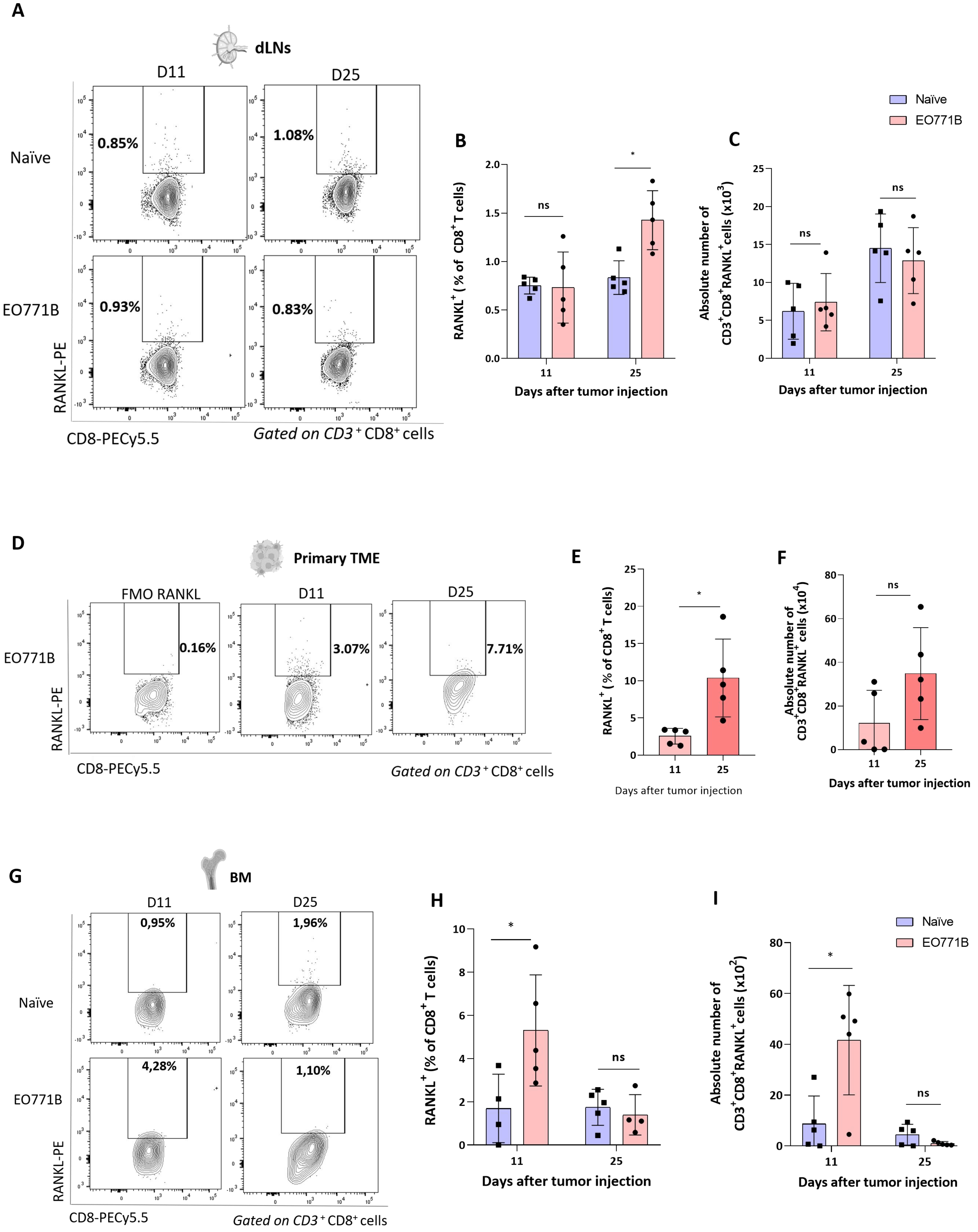
A
dLNs
D11
D25
Naïve
0.85%
1.08%
EO771B
0.93%
0.83%
RANKL-PE
CD8-PECy5.5
Gated on CD3+ CD8+ cells
B
RANKL+ (% of CD8+ T cells)
ns
*
11
25
Days after tumor injection
C
Absolute number of CD3+CD8+RANKL+cells (x10^3)
ns
ns
Naïve
EO771B
D
Primary TME
FMO RANKL
0.16%
3.07%
7.71%
E
F
Absolute number of CD3+CD8+RANKL+ cells (x10^4)
G
BM
0,95%
1,96%
4,28%
1,10%
H
RANKL+ (% of CD8+ T cells)
I
Absolute number of CD3+CD8+RANKL+cells (x10^2)

## Supplementary Figure S10

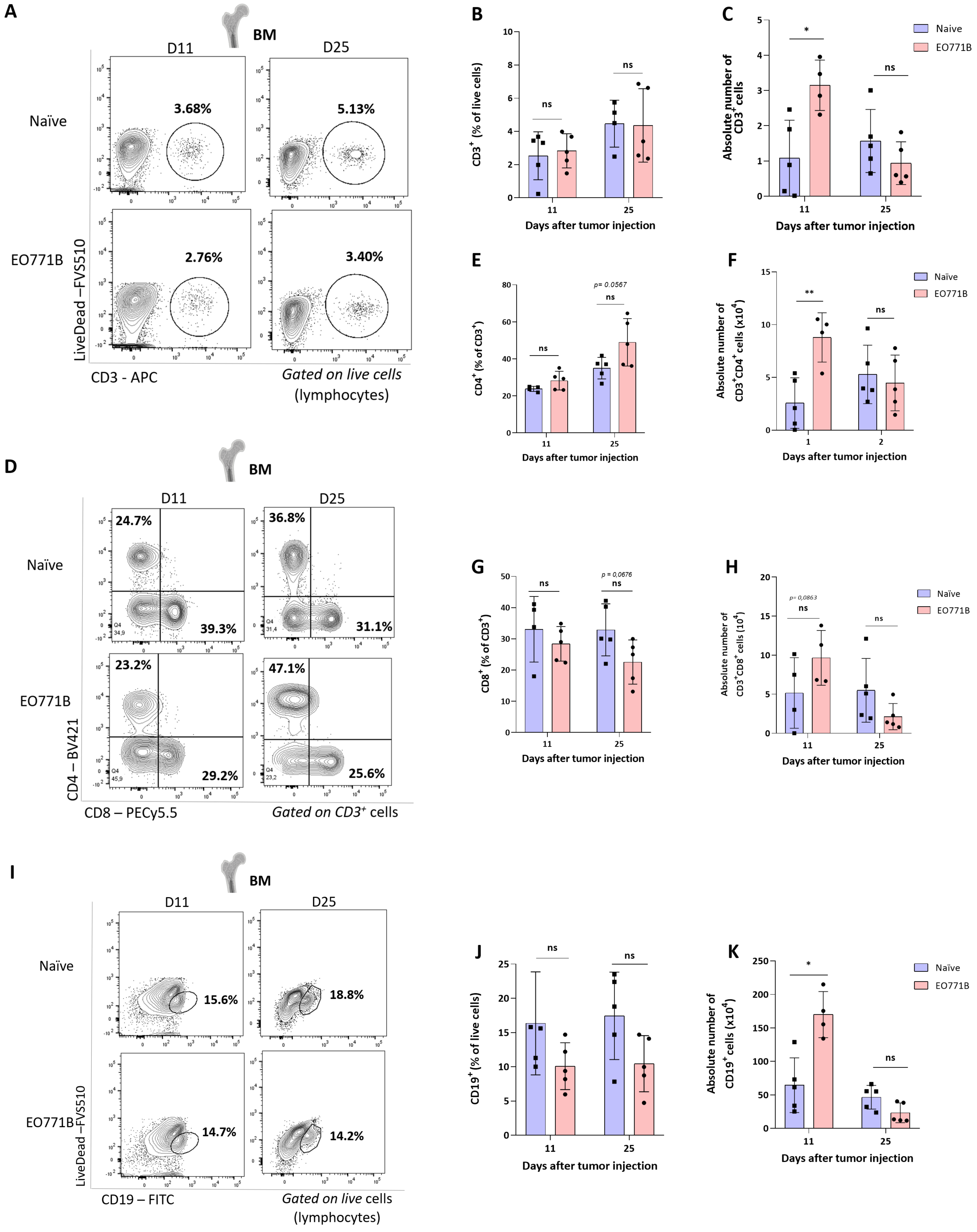

**Supplementary Figure S11**

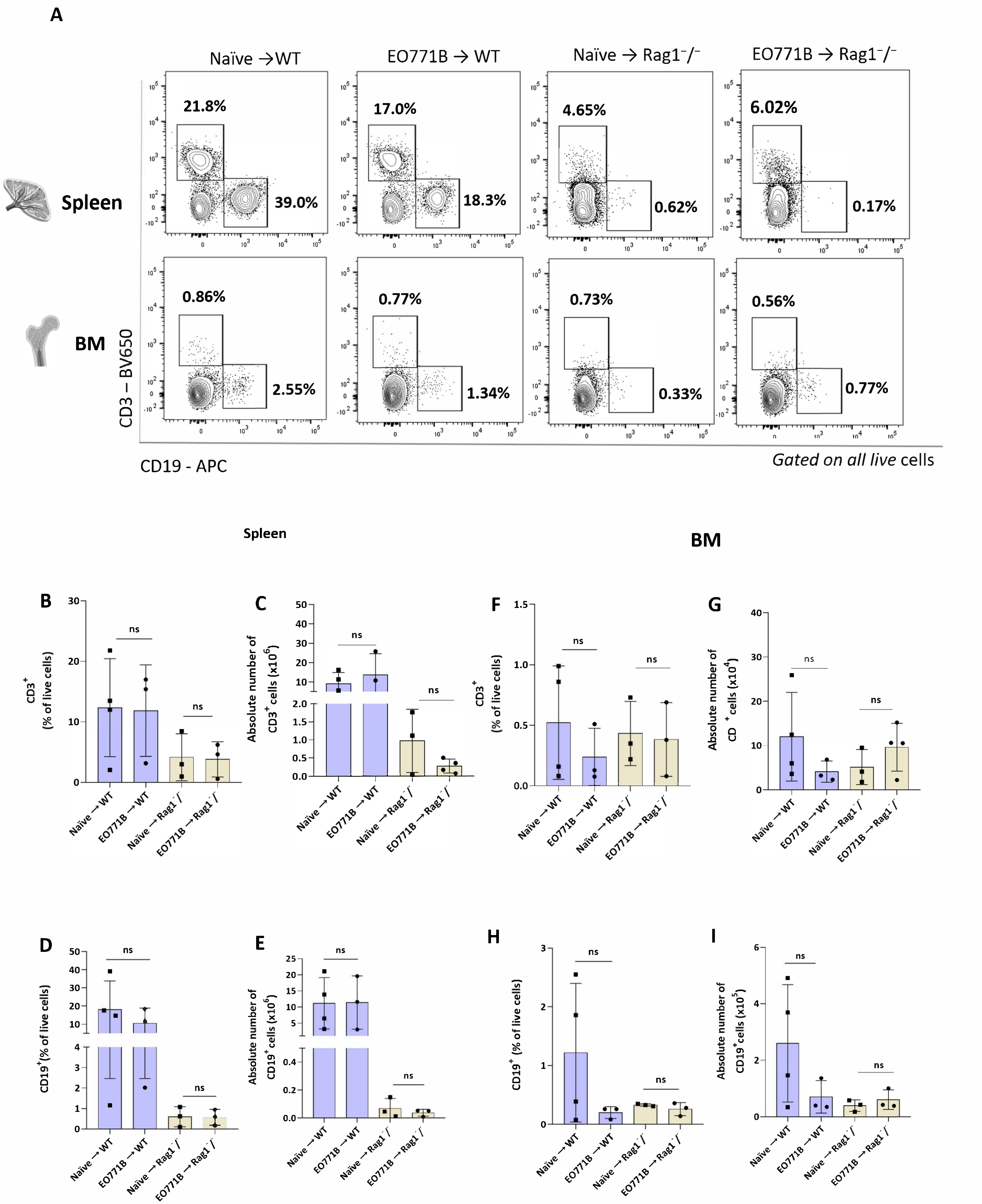

## Supplementary Figure S12

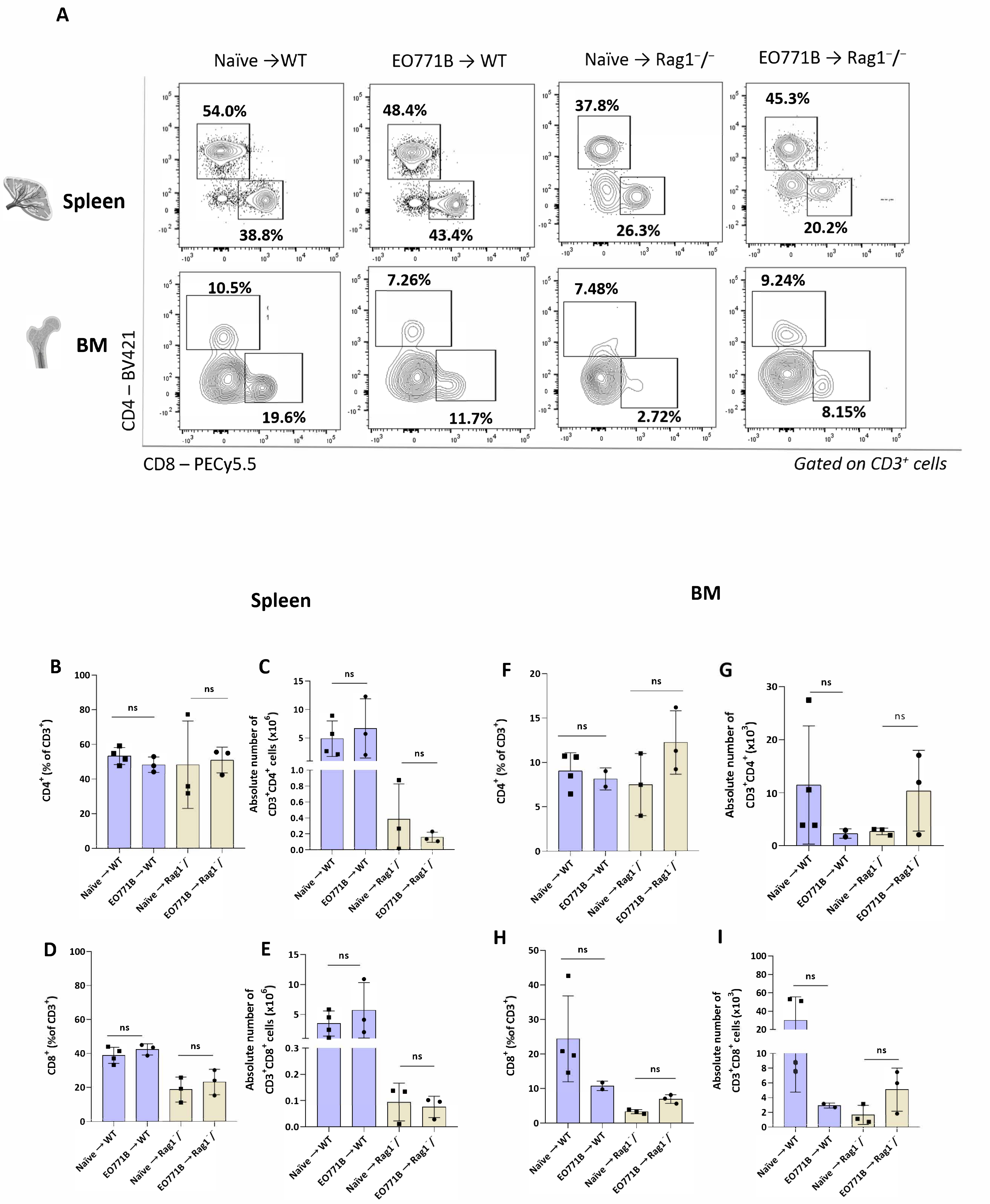

# Supplementary Figure S13

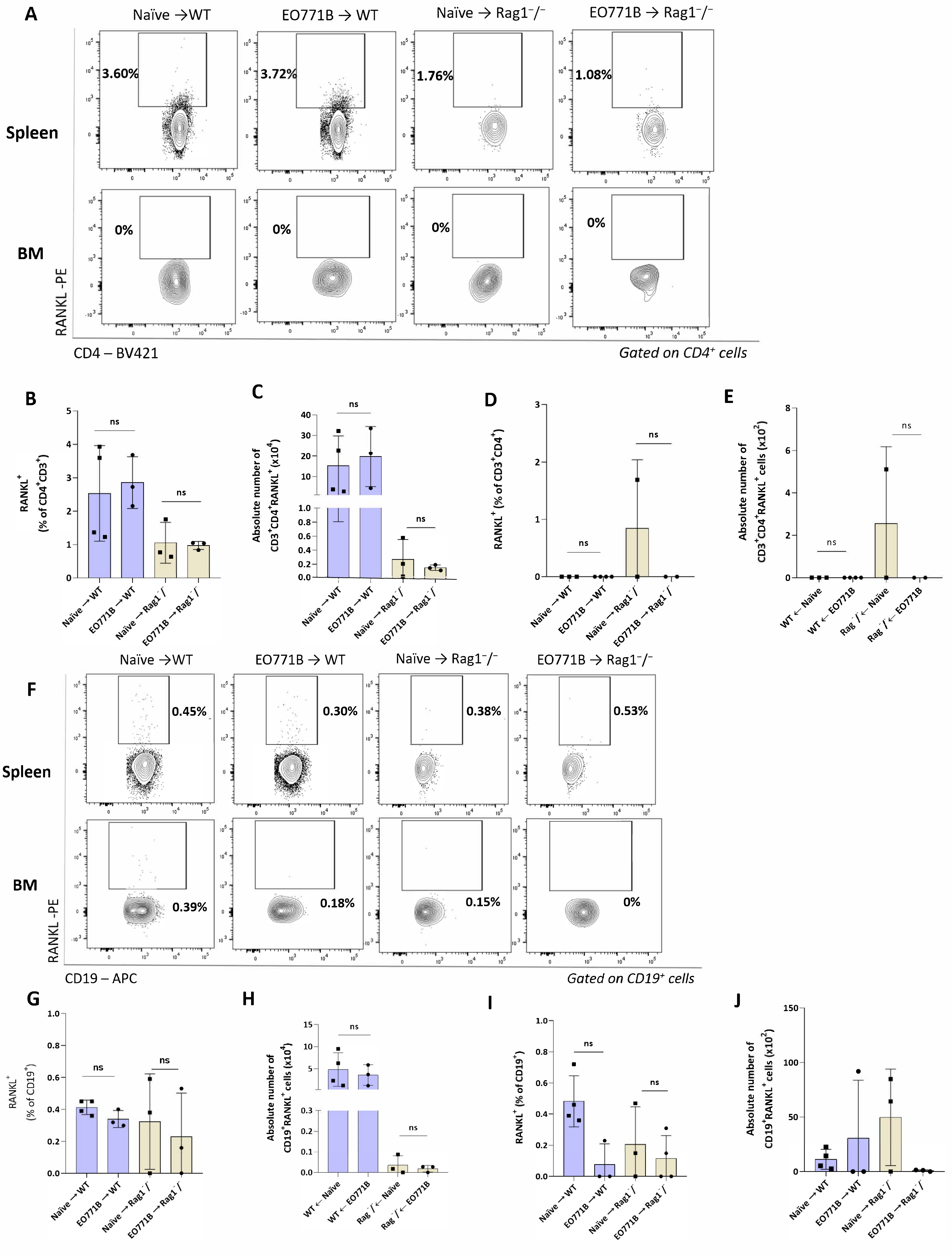

## Antibodies used for flow cytometry

| Antigen | Reactivity | Clone | Supplier | Fluorochrome | Dilution |
|---|---|---|---|---|---|
| **CD16/32** | Anti-mouse | 63 | Biolegend | - | 1:250 |
| **CD3** | Anti-mouse | 17A2 | ebioscience | APC | 1:100 |
| **CD4** | Anti-mouse | GK1.5 | BD | BV421 | 1:200 |
| **CD8** | Anti-mouse | 53-6.7 | ebioscience | PECy5.5 | 1:400 |
| **CD19** | Anti-mouse | 1D3 | BD | FITC | 1:100 |
| **FoxP3** | Anti-mouse | FJK-16S | ebioscience | PECy7 | 1:80 |
| **RANKL** | Anti-mouse | IK22-5 | BD | PE | 1:50 |
| **CD3e** | Anti-mouse | 17A2 | BD | BV650 | 1:200 |
| **CD19** | Anti-mouse | 1D3 | BD | APC | 1:200 |

**Supplementary Table S2**

| Figure panel | Bone parameter | Comparison | p value | Significance |
|---|---|---|---|---|
| **2B** | Bone volume (BV) | Naïve vs. EO771B D11 | 0.6844 | ns |
| **2B** | Bone volume (BV) | Naïve vs. EO771B D25 | 0.2586 | ns |
| **2B** | Bone volume (BV) | EO771B D11 vs. D25 | 0.1141 | ns |
| | | | | |
| **2C** | Bone volume fraction (BV/TV) | Naïve vs. EO771B D11 | 0.0067 | ** |
| **2C** | Bone volume fraction (BV/TV) | Naïve vs. EO771B D25 | 0.0013 | ** |
| **2C** | Bone volume fraction (BV/TV) | EO771B D11 vs. D25 | 0.0872 | ns |
| | | | | |
| **2D** | Bone surface area (BS) | Naïve vs. EO771B D11 | 0.0179 | * |
| **2D** | Bone surface area (BS) | Naïve vs. EO771B D25 | 0.0011 | ** |
| **2D** | Bone surface area (BS) | EO771B D11 vs. D25 | 0.3995 | ns |
| | | | | |
| **2E** | Trabecular separation (Tb.Sp) | Naïve vs. EO771B D11 | 0.1511 | ns |
| **2E** | Trabecular separation (Tb.Sp) | Naïve vs. EO771B D25 | 0.0500 | * |
| **2E** | Trabecular separation (Tb.Sp) | EO771B D11 vs. D25 | 0.0880 | ns |
| | | | | |
| **2F** | Trabecular thickness (Tb.Th) | Naïve vs. EO771B D11 | 0.0061 | ** |
| **2F** | Trabecular thickness (Tb.Th) | Naïve vs. EO771B D25 | 0.0316 | * |
| **2F** | Trabecular thickness (Tb.Th) | EO771B D11 vs. D25 | 0.00015 | *** |
| | | | | |
| **2G** | Trabecular number (Tb.N) | Naïve vs. EO771B D11 | 0.0232 | * |
| **2G** | Trabecular number (Tb.N) | Naïve vs. EO771B D25 | 0.0173 | * |
| **2G** | Trabecular number (Tb.N) | EO771B D11 vs. D25 | 0.0308 | * |

**Supplementary Table S3**

| Figure panel | Comparison | p value | Significance |
|---|---|---|---|
| **Figure 3** | | | |
| **3B** | Naïve vs. EO771B D11 | 0.0050 | ** |
| **3B** | Naïve vs. EO771B D25 | 0.00016 | *** |
| **3C** | Naïve vs. EO771B D11 | 0.0237 | * |
| **3C** | Naïve vs. EO771B D25 | 0.0132 | * |
| **3E** | EO771B D11 vs. D25 | 0.1598 | ns |
| **3F** | EO771B D11 vs. D25 | 0.0500 | * |
| | | | |
| **Figure 4** | | | |
| **4B** | Naïve vs. EO771B D11 | 0.0009 | *** |
| **4B** | Naïve vs. EO771B D25 | 0.0123 | * |
| **4C** | Naïve vs. EO771B D11 | 0.0049 | ** |
| **4C** | Naïve vs. EO771B D25 | 0.0005 | *** |
| **4E** | EO771B D11 vs. D25 | 0.1909 | ns |
| **4F** | EO771B D11 vs. D25 | 0.0115 | * |
| | | | |
| **Figure 5** | | | |
| **5B** | Naïve vs. EO771B D11 | 0.0387 | * |
| **5B** | Naïve vs. EO771B D25 | 0.4507 | ns |
| **5C** | Naïve vs. EO771B D11 | 0.0475 | * |
| **5C** | Naïve vs. EO771B D25 | 0.9226 | ns |
| **5E** | Naïve vs. EO771B D11 | 0.0435 | * |
| **5E** | Naïve vs. EO771B D25 | 0.7377 | ns |
| **5F** | Naïve vs. EO771B D11 | 0.0305 | * |
| **5F** | Naïve vs. EO771B D25 | 0.2403 | ns |

**Supplementary Table 4**

| Bone parameter | Pairwise comparison | Tukey-p value | Significance |
|---|---|---|---|
| **BV/TV** | Naïve → WT vs. EO771B → WT | 0.000561 | *** |
| | Naïve → Rag1$^{-/-}$ vs. EO771B → Rag1$^{-/-}$ | 0.011085 | * |
| | Untreated Rag1$^{-/-}$ vs. Naïve → Rag1$^{-/-}$ | 0.910227 | ns |
| | Untreated Rag1$^{-/-}$ vs. EO771B → Rag1$^{-/-}$ | 0.005842 | ** |
| | | | |
| **Tb.Th** | Naïve → WT vs. EO771B → WT | 0.002125 | ** |
| | Naïve → Rag1$^{-/-}$ vs. EO771B → Rag1$^{-/-}$ | 0.000498 | *** |
| | Untreated Rag1$^{-/-}$ vs. Naïve → Rag1$^{-/-}$ | 0.988073 | ns |
| | Untreated Rag1$^{-/-}$ vs. EO771B → Rag1$^{-/-}$ | 0.000690 | *** |
| | | | |
| **Tb.Sp** | Naïve → WT vs. EO771B → WT | 0.000541 | *** |
| | Naïve → Rag1$^{-/-}$ vs. EO771B → Rag1$^{-/-}$ | 0.002036 | ** |
| | Untreated Rag1$^{-/-}$ vs. Naïve → Rag1$^{-/-}$ | 0.329994 | ns |
| | Untreated Rag1$^{-/-}$ vs. EO771B → Rag1$^{-/-}$ | 0.154342 | ns |
